# DOCKSTRING: Easy Molecular Docking Yields Better Benchmarks for Ligand Design

**DOI:** 10.1021/acs.jcim.1c01334

**Published:** 2022-07-18

**Authors:** Miguel García-Ortegón, Gregor N. C. Simm, Austin J. Tripp, José Miguel Hernández-Lobato, Andreas Bender, Sergio Bacallado

**Affiliations:** † Statistical Laboratory, Centre for Mathematical Sciences, University of Cambridge, Wilberforce Rd., Cambridge CB3 0WB, United Kingdom; ‡ Department of Engineering, University of Cambridge, Trumpington St., Cambridge CB2 1PZ, United Kingdom; ¶ Yusuf Hamied Department of Chemistry, University of Cambridge, Lensfield Rd., Cambridge CB2 1EW, United Kingdom

## Abstract

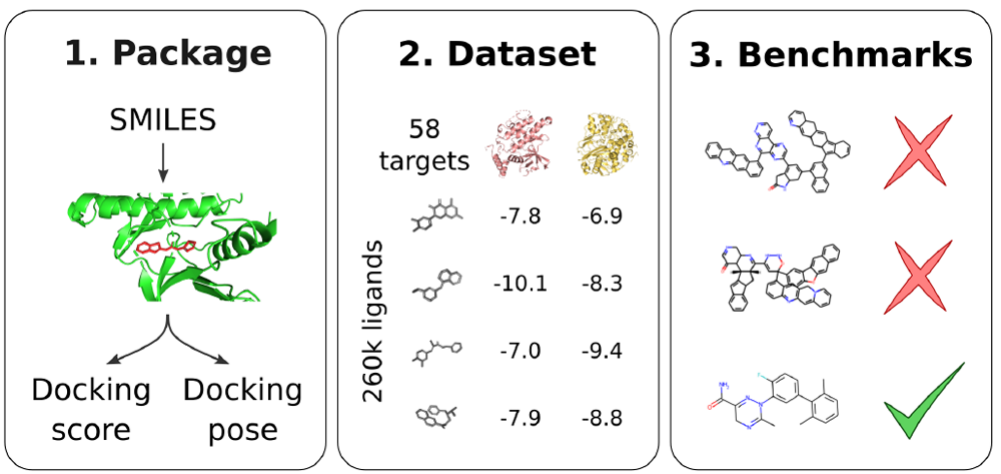

The field of machine learning for drug discovery is witnessing an explosion of novel methods. These methods are often benchmarked on simple physicochemical properties such as solubility or general druglikeness, which can be readily computed. However, these properties are poor representatives of objective functions in drug design, mainly because they do not depend on the candidate compound’s interaction with the target. By contrast, molecular docking is a widely applied method in drug discovery to estimate binding affinities. However, docking studies require a significant amount of domain knowledge to set up correctly, which hampers adoption. Here, we present dockstring, a bundle for meaningful and robust comparison of ML models using docking scores. dockstring consists of three components: (1) an open-source Python package for straightforward computation of docking scores, (2) an extensive dataset of docking scores and poses of more than 260,000 molecules for 58 medically relevant targets, and (3) a set of pharmaceutically relevant benchmark tasks such as virtual screening or *de novo* design of selective kinase inhibitors. The Python package implements a robust ligand and target preparation protocol that allows nonexperts to obtain meaningful docking scores. Our dataset is the first to include docking poses, as well as the first of its size that is a full matrix, thus facilitating experiments in multiobjective optimization and transfer learning. Overall, our results indicate that docking scores are a more realistic evaluation objective than simple physicochemical properties, yielding benchmark tasks that are more challenging and more closely related to real problems in drug discovery.

## 1 Introduction

The field of industrial drug discovery is undergoing a crisis. Despite significant technological advances, R&D costs have grown by orders of magnitude, while the probability of success of candidate molecules has decreased. This phenomenon is partly attributed to a lack of sufficiently predictive experimental and computational models.^1^ Machine learning (ML) is widely regarded as a promising technology to tackle this issue by providing faster and more accurate models.^2^

The rapid development of ML methods for drug discovery^3,4^ has resulted in a growing need for high-quality benchmarks to allow for these methods to be evaluated and compared. Ideally, a good benchmark would test a model on accurate experimental data (e.g., experimental bioactivity data) in a realistic problem setting (e.g., prospective search), so that strong performance on the benchmark would imply strong performance on real-world tasks. Unfortunately, the high cost and difficulty of collecting experimental data makes such benchmarks impractical. Existing benchmarks tend to either (1) use a fixed experimental dataset for problem settings like in-distribution regression^5^ or (2) use simple computational properties for problem settings like *de novo* design. The latter type of benchmark is popular in the ML community with the tasks of maximizing the quantitative estimate of druglikeness (QED)^6^ and penalized log partition coefficient (logP) being highly prevalent.^7−14^ However, the simplicity of these properties raises doubts about whether performance on such benchmarks is indicative of performance on real drug-design tasks.

Previous works have suggested that molecular docking could form the basis for high-quality benchmarks.^15−17^ Molecular docking is a computational technique that attempts to predict how a small molecule (the *ligand*) binds to a protein receptor (the *target*) by simulating the physical interaction between the two.^18^ The output of this simulation is a *docking score*, which estimates the strength of binding between the molecule and protein, and a *docking pose*, the predicted 3D conformation of the ligand in the protein binding pocket. Below, we summarize some of the benefits of molecular docking over simple physicochemical properties (e.g., logP) with regard to benchmarking:

(1) Interpretability: docking scores have a structural interpretation in terms of predicted binding affinity,^19^ correlating with experimental values in some protein families.^20^
(2) Relevance: docking scores are routinely employed by medicinal chemists in academia and industry to discover hits in virtual screening experiments. Docking poses are also used to identify and exploit important interactions during lead optimization.
(3) Computational cost: docking scores can typically be computed in under a minute, unlike other computational methods like free energy perturbation calculations or density functional theory.
(4) Challenging benchmark: the relationship between molecular structure and docking score is complex, as the docking score depends on the 3D structure of the ligand–target complex. Therefore, tasks based on docking require ML models to learn complex 3D features.

Because of these advantages, it is unsurprising that several recent works have applied ML to tasks based on docking scores.^21−25^ Yet, there are still several hurdles which make a docking benchmark difficult to realize. First, such a benchmark mandates high-quality standardization. Running a docking simulation involves many intermediate steps, such as target and ligand preparation and the specification of a *search box*. Each step requires significant domain expertise, and for a benchmark to facilitate a meaningful comparison between algorithms, they must be carried out correctly and consistently. Second, the benchmark needs to be accessible to those without domain knowledge. Finally, the benchmark needs to contain breadth and diversity of targets.

A fully automated docking software pipeline is a potential way to overcome these hurdles. Indeed, there are several existing works which try to facilitate the use of molecular docking for ML benchmarks. However, these works all lack at least one of the aforementioned desiderata. VirtualFlow^26^ and DockStream^27^ (part of the REINVENT ecosystem^28^) are general-purpose wrappers for docking programs. However, they primarily cater to docking experts requiring manually prepared target files and specialized arguments. The therapeutics data commons (TDC)^17^ and Cieplinski et al.^16^ provide wrappers which offer computation of docking scores from just a SMILES string. However, both wrappers have shortcomings with respect to standardization. Neither TDC nor Cieplinski et al. control sources of randomness during the docking procedure (e.g., random seeds input into the docking program or the conformer generation routines), leading to the potential for considerable variance between runs on the same molecule. Further, at the time of this writing, both wrappers have a relatively rudimentary ligand preparation pipeline; for example, neither of them perform ligand protonation, an important part of the preparation process.^29,30^ Moreover, both of these wrappers provide only a small number of targets: TDC provides only one target, while Cieplinski et al.^16^ provide just four.

In addition to wrappers, several docking benchmarks have been developed. The Directory of Useful Decoys Enhanced (DUD-E)^31^ is a relatively small ligand set of actives and property-matched decoys for 102 targets. Originally designed to evaluate docking algorithms, its ligand set has since been widely applied to benchmark ML models for virtual screening.^32−34^ However, it has been shown that DUD-E is easily overfit by ML models, which are able to memorize actives and decoys.^35,36^ Therefore, using DUD-E as a benchmark for virtual screening will likely lead to an overestimation of performance. The evaluation framework GuacaMol^37^ provides both a distribution matching and goal-directed benchmark suite, with the latter containing 20 distinct tasks based on molecular fingerprints, substructure matching, and physicochemical properties. Although most of these tasks are challenging, they are largely based on simple physicochemical properties and similarity functions such as the Tanimoto similarity. As a result, they fail to capture subtleties related to 3D molecular structure or interactions with biomolecules. The benchmark suite MOSES^38^ provides several molecular generation benchmarks that focus on generating a diverse set of molecules rather than optimizing for any particular chemical property. MoleculeNet^5^ is a large compilation of datasets for benchmarking regression and classification with ML models. It includes medically relevant end points such as blood–brain barrier penetration or phenotypic toxicity screens. However, overlap between datasets is not guaranteed, and there is no option to compute new labels, which makes it challenging to evaluate transfer learning or *de novo* design.

**Figure 1:**
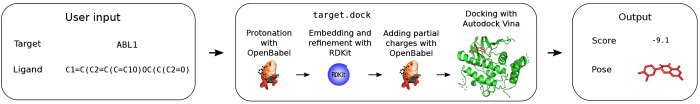
Summary of dockstring pipeline from SMILES strings to scores and poses. The method target.dock performs ligand preparation with Open Babel and RDKit and docking with AutoDock Vina.

In this work, we introduce dockstring, a bundle for standardized and accessible benchmarking of ML models based on molecular docking. It consists of three parts: a Python package for easy computation of docking scores, a large and diverse dataset of docking scores and poses for pretraining, and a set of meaningful benchmark tasks on which to evaluate models (Figure 1).

(1) Python package: a user-friendly Python wrapper of the popular docking package AutoDock Vina^39^ (Sections 2.1 and 3.1). AutoDock Vina was selected due to its high-quality docking poses, reasonable accuracy of predicted binding free energies, and low computational cost.^20,40^ The emphasis of our package is on simplicity—a full docking calculation can be set up in just four lines of code.
(2) Dataset: a dataset of over 260,000 diverse and druglike molecules docked against a curated list of 58 targets, resulting in more than 15 million docking scores and poses (Sections 2.2 and 3.2). The high number of activity labels per molecule makes our dataset highly suitable for the design of meaningful benchmark tasks in ML settings such as multiobjective optimization or transfer learning. Furthermore, targets are selected to represent a number of protein families of high pharmaceutical value, such as kinases or nuclear receptors. Overall, more than 500,000 CPU hours were invested in the creation of the dataset.
3. Benchmarks: a set of pharmaceutically relevant and challenging benchmark tasks covering regression, virtual screening, and *de novo* design (Sections 2.3 and 3.3).

## 2 Methods

### 2.1 Python Package

#### 2.1.1 Target Preparation

There are 58 prepared targets available in dockstring. PDB files of 57 protein targets were downloaded from the Directory of Useful Decoys Enhanced (DUD-E), a database of proteins and ligands for benchmarking docking algorithms.^31^ Structures in DUD-E were determined experimentally to high precision, with the large majority of resolutions being less than 2.5 Å. Furthermore, DUD-E targets were prepared to improve correlation between theoretical and experimental binding affinities. For instance, in a few cases, the authors of DUD-E manually added cofactors or crystallographic waters or changed the protonation states of side residues.^31^ For dockstring, the PDB files were standardized with Open Babel^41^ (e.g., the symbols of some metal atoms were not recognized by AutoDock Tools^42^). Polar hydrogen atoms were added, and conversion to the PDBQT file format was carried out with AutoDock Tools.^42^

The only target that does not originate from DUD-E is DRD2, the dopamine receptor D2. It was included in dockstring due to its popularity in molecular regression and optimization.^43−47^ To ensure consistency, the preparation of DRD2 was analogous to that of its homologue DRD3 in DUD-E. Starting from a crystal structure of DRD2 (PDB 6CM4),^48^ the bound inhibitor (risperidone) as well as residual water and solute molecules were manually removed with PyMOL,^49^ since DRD3 in DUD-E did not include any waters or ions. Subsequently, the structure was optimized with the program obminimize from Open Babel using the general Amber force field (GAFF).^50^ Protonation was carried out at pH 7.4 with PROPKA.^51^ Finally, addition of polar hydrogen atoms and conversion to PDBQT were performed with AutoDock Tools.

The search box of each target in dockstring was also determined. Every DUD-E structure has a corresponding ligand file from which the box position and size were derived. We computed the maximum and minimum coordinates of each ligand across each dimension and padded with 12.5 Å on all sides. Finally, if a box length did not reach 30 Å after padding, we set it to this amount. The padding length and the minimum box length were tuned manually to minimize the number of positive scores, which indicate highly constrained poses. The search box of DRD2 was set manually upon visual examination of the binding pocket in the reference structure bound to risperidone.^48^

#### 2.1.2 Ligand Preparation

Ligands are provided to the dockstring package as SMILES strings. First, dockstring performs a sanity check on the ligand. Ligands with radicals or ligands consisting of more than one molecular fragment are rejected. Next, the ligand is (de)protonated at pH 7.4 with Open Babel.^41^ While automated protonation protocols are not perfect,^29^ their application is widely regarded as good practice.^30^ Then, a single 3D conformation is generated with the Euclidean distance geometry algorithm ETKG^52^ as implemented in RDKit.^53^ This conformation is subsequently refined with the classical force field MMFF94.^54^ During the embedding of the graph representation into a 3D structure, the stereochemistry of determined stereocenters is maintained, whereas any undetermined stereocenters are assigned randomly (but consistently across different runs to ensure the reproducibility of docking scores). Finally, dockstring computes the Gasteiger charges^55^ for all atoms and creates a ligand PDBQT file with Open Babel.

#### 2.1.3 Molecular Docking with AutoDock Vina

dockstring docks a ligand against a target using AutoDock Vina.^39^ The ligand PDBQT input file is obtained automatically as explained in Section 2.1.2, while the target PDBQT and search boxes are taken from the list of prepared input files as explained in Section 2.1.1. Docking is performed with the default values of exhaustiveness (8), maximum number of binding modes (9), and energy range (3). After docking is complete, we obtain up to nine poses, together with their binding free energies. Note that in subsequent analyses we only use the lowest docking score (i.e., the best one).

Ligand preparation and molecular docking, and thus the docking score, depend on a random seed. We investigated this dependence and found no target–ligand combination for which the docking scores deviated by more than 0.1 kcal/mol. Subsequently, we fixed the random seed to obtain a fully deterministic pipeline.

### 2.2 Dataset

#### 2.2.1 Target and Ligand Selection

As explained in Section 2.1.1, most targets originate from DUD-E,^31^ a database of proteins and ligands for comparison and development of docking algorithms. These targets are medically relevant and cover a large variety of protein families, functions, and structures. We only selected targets with more than 1000 experimental actives in ExCAPE (see below) to ensure a high number of positive examples. In addition to the targets from DUD-E, we also included the target DRD2, a popular benchmark in ML.^43−47^

Ligand molecules and activity labels were taken from ExCAPE,^56^ a database that curates bioactivity assays from PubChem^57^ and ChEMBL.^58^ In turn, ExCAPE inherits labels from the original assays in PubChem and ChEMBL. The exact numeric threshold for actives and inactives may vary from one assay to another depending on the authors’ experience with the experimental protocol and the protein target. However, ExCAPE sets a normalizing constraint to remove every active with an affinity value above 10 μm.

We selected all ExCAPE molecules with active labels against the proteins in our target set (at least 1000 actives for each target, see above) and added another 150,000 molecules with inactive labels only. Experimental actives are more likely to receive good scores in docking simulations (i.e., scores that are negative in sign and large in absolute value), whereas experimental inactives are more likely to receive poor docking scores. Therefore, by combining experimental actives and inactives in our dataset, we expected to create a strong signal that facilitated supervised learning. After discarding 1.8% of molecules due to failures in the ligand preparation process, the final dataset consisted of 260,155 compounds.

#### 2.2.2 Clustering and Scaffold Analysis

In order to evaluate the diversity of our ligand set, we carried out a clustering analysis with DBSCAN (Density-Based Spatial Clustering of Applications with Noise) as implemented in scikit-learn.^59^ For this analysis, molecules were represented as RDKit fingerprints of path length six. The choice of the fingerprints was motivated by previous analysis by Landrum,^60^ suggesting that this type of fingerprint was the most appropriate for similarity search. The neighborhood cutoff ε was set to a Jaccard distance of 0.25.^61^ We also performed Bemis–Murcko scaffold decomposition, a classical form of clustering based on molecular graphs.^62^ Bemis–Murcko decomposition reduces each molecule to a simpler version of itself, called a scaffold, which consists of its ring systems and the linker atoms between them. A ring system is defined as either a ring or two or more rings sharing an edge. Furthermore, any atoms in the scaffold other than carbon are substituted by carbon. Molecules with the same scaffold are structurally similar and are expected to have similar properties, so they can be grouped into a single cluster. We implemented Bemis–Murcko scaffold decomposition with RDKit, using the function rdkit.Chem.Scaffolds.MurckoScaffold.GetScaffoldForMol.

### 2.3 Benchmarks

dockstring’s combination of a docking package and large dataset allows it to underpin a wide variety of benchmark tasks for supervised learning, active learning, transfer learning, meta-learning, molecule optimization, and more. We formulate benchmark tasks for three problem settings: regression, virtual screening, and *de novo* design (Table 1). The regression benchmark (Sections 2.3.1 and 3.3.1) is relatively standard and widely applicable; it primarily illustrates the difficulty of predicting docking scores. Virtual screening (Sections 2.3.2 and 3.3.2) evaluates a model’s ability to select active molecules from a large predefined library. This is a common use case for predictive models in the pharmaceutical industry and requires strong out-of-distribution performance to be successful. It is applicable to any method that can rank a list of molecules, either by regression or by other means. *De novo* design (Sections 2.3.3 and 3.3.3) evaluates the ability to generate novel molecules that optimize an objective function. It is an active area of research because chemical space is vast (more than 10^60^ by some estimates^63^), so even the largest libraries cover just a tiny fraction of it. Models for *de novo* design include optimization algorithms, reinforcement learning agents, or generative models. The objective functions presented here are all based on docking scores but vary in difficulty.

**Table 1:** Overview of Benchmarks Tasks in the dockstring Bundle

Setting	Description	Motivation	Proteins	Metric
Regression	Predict docking scores and minimize prediction error on held-out test set	Evaluation of molecular representations and predictive models	PARP1, F2, KIT, ESR2, PGR	Coefficient of determination (*R*^2^)
Virtual screening	Rank molecules according to their docking score and compute enrichment in top-*k*-ranking molecules	Model evaluation for hit discovery in large molecular libraries	PARP1, KIT, PGR	Enrichment factor (EF)
*De novo* design	Given a training dataset and a fixed budget of objective function evaluations, propose molecules that optimize an objective	Model evaluation for hit discovery by *de novo* molecular design	F2	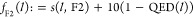
			PPAR{A, D, G}	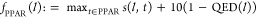
			JAK2, LCK	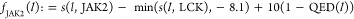

#### 2.3.1 Regression

##### Task Description

For each target, the task is to train a regression model to predict the docking score of a given SMILES string. The models were trained and tested on the dockstring dataset, split into training and test sets according to the cluster labels (Section 3.2.2). Cluster splitting is recommended because chemical datasets contain many analogous (yet unique) molecules, such that simple random split will likely lead to an overestimation of test performance.

##### Proposed Benchmark

While all targets could be used in this benchmark, the large number of targets in our dataset would make this benchmark expensive and difficult to interpret. Therefore, we selected five targets from different protein families whose docking scores were deemed of high quality, based on enrichment analysis of experimental activity labels (Section 3.2.1). To ensure that we included a range of difficulties, we performed an initial experiment where we regressed the docking scores of all high-quality targets. We found that performance varied considerably depending on the target and the method employed, with coefficients of determination *R*^2^ ranging between 0.2 and 0.9 (details in Table S3 of the Supporting Information (SI)). On the basis of these results, we proposed the five following benchmark targets (with protein function and level of difficulty in brakets): PARP1 (enzyme, easy), F2 (protease, easy to medium), KIT (kinase, medium), ESR2 (nuclear receptor, hard), and PGR (nuclear receptor, hard).

#### 2.3.2 Virtual Screening

##### Task Description

The goal of screening is to identify actives from a large library that is too big for detailed experimental analysis. In virtual screening, we attempt to solve this issue by scoring the library computationally and selecting a smaller subset with high scores. Then, this subset can be studied in detail.^64^ A classical metric to evaluate the effectiveness of screening methods is the enrichment factor (EF), which is defined as the rate of actives in the selected subset over the rate of actives in the initial library.^65^ Intuitively, a high enrichment factor indicates that undesirable compounds are deprioritized by the screening method, thus allowing us to focus our limited resources on a smaller set that is enriched in actives.

Here, we propose a task to benchmark virtual screening ML models using docking scores as ground truth. The model must rank all compounds in the ZINC20 database according to their predicted docking score. ZINC20 contains around 1 billion commercially available druglike molecules.^66,67^ Then, the docking scores of the top-ranking subset are computed with the dockstring package. Molecules with a score better (i.e., lower) than a certain threshold are labeled as actives. Finally, the enrichment factor (EF) of the top subset is computed. We chose the threshold to be the lowest 0.1 percentile of the ZINC20 database, which we estimated from a random sample of 100,000 molecules. Therefore, the rate of actives before virtual screening was 10^–3^, and the maximum possible enrichment in our benchmark is 10^3^.

Note that since our benchmark uses docking scores as ground truth, the applicability of benchmarked models to real-world binding problems will depend on the applicability of the underlying docking scores. Therefore, our virtual screening task should not be viewed as a substitute to real-world screening itself. Rather, it can be used as a realistic evaluation to guide the development of high-performing models for virtual screening. Then, once high-performing models have been identified, they can be trained on experimental data to make them applicable to real-world problems.

Also note that even though both the virtual screening benchmark and the regression benchmark involve some type of prediction of docking scores, they are very different settings. First, virtual screening only requires *ranking* compounds by score rather than explicitly predicting the scores’ numeric values. Second, the evaluation metric is not the same. The enrichment factor (EF), which is popular in chemoinformatics but uncommon in ML, only considers the top molecules, whereas regression metrics such as the coefficient of determination *R*^2^ evaluates all molecules.

##### Proposed Benchmark

We trained models on the docking scores of PARP1, KIT, and PGR using all molecules in our dataset. As in the regression benchmark, these targets were chosen to represent a range of regression difficulties. Trained models were used to rank all the molecules in ZINC20 and select the top 5000 compounds with the lowest predicted scores. Once the most promising molecules had been selected, we computed their actual docking scores with dockstring. Molecules were labeled as active if their actual scores were below the 0.1 percentile threshold, which was −10.7 for KIT, −12.1 for PARP1, and −10.1 for PGR. Finally, the enrichment factor (EF) was computed as the ratio of active molecules in the selected subset over the ratio of active molecules in ZINC20 (which was 0.1% by design; see above).

#### 2.3.3 *De Novo* Molecular Design

##### Task Description

The goal of *de novo* design is to propose novel molecules that optimize an objective function given a certain budget. To be representative of real problems in drug discovery, this budget should be high enough to allow for significant exploration but small enough to resemble the experimental budget of a committed wet lab.

Docking scores are biased toward high molecular weight^68^ and lipophilicity. Therefore, optimizing docking scores alone can lead to large and hydrophobic molecules, as we observed in our initial experiments (Figure 9). These compounds are undesirable because they will suffer from poor ADMET properties and off-target effects.^68,69^ We found that adding a druglikeness penalty based on QED helped remedy this issue.

##### Proposed Benchmark

The goal of each *de novo* design task is to minimize a docking-based objective function, having access to the whole dataset and 5000 function evaluations. In the case of predictive generative models such as GP-BO, the whole dataset could be used to learn the docking score function, whereas in genetic algorithms, it could be used to set the initial population. We propose three objective functions, all of which contain a weighted QED term to promote druglikeness. Let *t* be a target,  be a ligand, and *s*(, *t*) be the docking score of  against *t*. Let QED() be the QED value of .

1. **F2**: a comparatively easy task that requires docking well to a single protein.

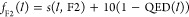
1

2. **Promiscuous PPAR**: requires strong binding to the three PPAR nuclear receptors. PPAR scores are positively correlated, so this is a task of medium difficulty. “Promiscuous” pan-PPAR agonists are being researched as treatments against metabolic syndrome.^70^ If PPAR := {PPARA, PPARD, PPARG}, then the objective function is

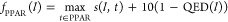
2

3. **Selective JAK2**: requires strong binding to JAK2 and weak binding to LCK. The challenge is that, since they are both kinases, their scores are positively correlated (ρ = 0.80). Due to their role in cell signaling and cancer, kinases are highly relevant targets. However, achieving selectivity is notoriously difficult, and off-target effects and toxicity are common.^71^ Our proposed objective anchors the LCK score to its median (− 8.1)

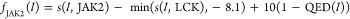
3

### 2.4 Baselines

We tested a variety of classical and more modern algorithms to assess the difficulty of the dockstring benchmarks tasks. Training and testing datasets and procedures followed each tasks’ specifications as described in Section 2.3. Additional details are given in the SI.

#### 2.4.1 Regression and Virtual Screening

##### scikit-learn Algorithms

Ridge and lasso regression were implemented with scikit-learn. XGBoost was implemented with the XGBoost library^72^ using the scikit-learn API. For all these methods, hyperparameter selection was done with random search over 20 configurations, evaluating each configuration using a five-fold cross-validation score (implemented via scikit-learn’s RandomizedSearchCV function).

##### Gaussian Processes

All Gaussian process (GP) algorithms used the Tanimoto kernel^73^ with fingerprint features. Due to the cubic scaling of GP regression, the exact GP was trained on 10,000 randomly chosen data points. In comparison, the sparse GP used 10,000 randomly chosen training points as the inducing variables but was trained on the whole dataset. Hyperparameters were chosen by maximizing the log-marginal likelihood on the training set. All GPs were implemented with PyTorch^74^ and GPyTorch.^75^

##### Graph Neural Networks

The DeepChem library’s implementation of the Attentive FP and MPNN was used.^76^ Both models were trained with default parameters from the DeepChem library for 10 epochs. Preliminary experiments with a third method from DeepChem, the Graph Attention Network,^77^ were performed but the model frequently overfitted to the training data; we decided to omit it rather than tune the hyperparameters for this model.

##### Fingerprint Similarity Search

To compare ML models with a classical virtual screening method, we added fingerprint similarity search to our baselines. Molecules were represented with binary RDKit fingerprints of path length 6, and similarities were computed as Tanimoto similarities. Given a query molecule from the ZINC dataset, we found the closest molecule in the dockstring dataset and copied its activity label. Note that active and inactive labels were not derived from experimental data; rather, they had been assigned according to a docking score cutoff set to the lowest 0.1 percentile of the score distribution (Section 2.3.2). ZINC molecules that were labeled inactive were discarded, and those that were labeled active were sorted by similarity to the closest neighbor in dockstring. Finally, the top 5000 molecules (i.e., the 5000 that were closest to a dockstring active) were selected for evaluation (and computation of the enrichment factor).

#### 2.4.2 *De Novo* Design

##### Graph Genetic Algorithm

The implementation from the GuacaMol baselines^37^ was used.^78^ The population size was set to 250, the offspring size to 25, and the mutation rate to 0.01. The population size was chosen based on some preliminary experiments with the GuacaMol dataset, and the offspring size was arbitrarily chosen to be 25 to allow for 200 generations to occur. The value of the mutation rate was the default used in the GuacaMol implementation.

##### SELFIES Genetic Algorithm

The implementation of the SELFIES genetic algorithm was taken from the GitHub repository of Nigam et al.^79^ It is a simple genetic algorithm which randomly inserts, deletes, or modifies a single token of a SELFIES string.^80^ The algorithm was not tuned and represents the minimum level of performance that can be expected from any reasonable genetic algorithm. The offspring and population size hyperparameters were the same as for the graph genetic algorithm.

##### Bayesian Optimization

The GP implementation is identical to the exact GP implementation from Section 3.3.1 using the Tanimoto kernel.^73,81^ As it is computationally infeasible to train a GP on the entire dataset, the 2000 training points with the smallest objective score and 3000 random points were selected from the dataset for training. Kernel hyperparameters were chosen by maximizing the log marginal likelihood on this training set. At each iteration, a batch of five new molecules was selected by maximizing either the upper confidence bound acquisition function^82^ with β = 10 (i.e., μ + 10σ) or the expected improvement acquisition function.^83^ β was chosen based on the GP hyperparameters from Section 3.3.1 and a small amount of preliminary experiments on the GuacaMol benchmarks to encourage a combination of exploration and exploitation but was not tuned once experiments on the docking objectives were started. Optimization was done using the graph genetic algorithm as described above, with an offspring size of 1000 and 25 generations. The batch was then scored and the GP retrained using the new scores, with the hyperparameters remaining unchanged. This was repeated until the objective function evaluation budget was reached.

## 3 Results

This section introduces the three components of the dockstring bundle: a user-friendly molecular docking package, an extensive dataset, and a set of challenging benchmark tasks. All three components are available at https://dockstring.github.io under the Apache 2.0 license.

### 3.1 Molecular Docking Package

**Figure 2:**
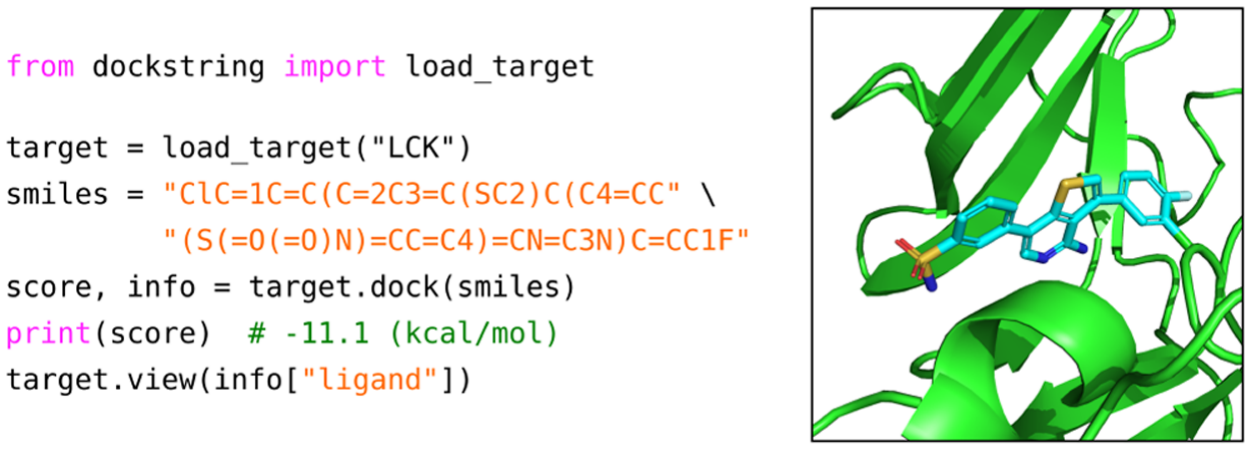
dockstring provides a simple API for docking and visualization. User-defined targets and custom pH’s can be specified if required. (Left) Code example for docking. (Right) Visualization of the docking pose in the active site of the target LCK.

We developed a Python package that interfaces with AutoDock Vina to allow the computation of docking scores in just a few lines of code. The user only needs to provide the name of a target protein and the SMILES string of a ligand molecule (Figure 2, left). The target name can be chosen from a list of 58 targets (Table 2) that have been prepared as explained in Section 2.1.1. Ligands are prepared automatically by the dockstring package as explained in Section 2.1.2. dockstring returns up to nine docking poses with their corresponding docking scores, which can be used in downstream tasks such as bioactivity prediction and visualization (Figure 2, right). Note that in subsequent experiments in this work, we always use the lowest (i.e., best) docking score. For most targets, computing a score with eight CPUs takes around 15s (Table S7 of the SI). We also note that, by default, our docking wrapper carefully controls all sources of randomness in the docking procedure so that the output is deterministic (Section 2.1.3). Finally, the target, the search box, and all poses can be visualized with the PyMOL software package.

**Table 2:** Targets in the dockstring Dataset Grouped by Function and Quality of Docking Scores^a^

Group	Quality	Gene
Kinase	***	IGF1R, JAK2, KIT, LCK, MAPK14, MAPKAPK2, MET, PTK2, PTPN1, SRC
	**	ABL1, AKT1, AKT2, CDK2, CSF1R, EGFR, KDR, MAPK1, FGFR1, ROCK1
	*	MAP2K1, PLK1
Enzyme	***	HSD11B1, PARP1, PDE5A, PTGS2
	**	ACHE, MAOB
	*	CA2, GBA, HMGCR, NOS1, REN, DHFR
Nuclear receptor	***	ESR1, ESR2, NR3C1, PGR, PPARA, PPARD, PPARG
	**	AR
	*	THRB
Protease	***	ADAM17, F10, F2
	**	BACE1, CASP3, MMP13
	*	DPP4
GPCR	**	ADRB1, ADRB2, DRD2, DRD3
	*	ADORA2A
Cytochrome	**	CYP2C9, CYP3A4
Chaperone	*	HSP90AA1

a ***: best. *: worst.

### 3.2 Dataset

Molecular docking is applicable in areas such as regression, molecular optimization, virtual screening, transfer learning, multitask learning, and representation learning. Since most of these settings require an initial training dataset, we provide a set of more than 15 million scores for a diverse and highly curated set of more than 260, 000 molecules docked against 58 targets. This dataset required more than 500,000 CPU hours to compute (see Section D of the SI for computational details). The target and ligand selection process are detailed below.

#### 3.2.1 Target Selection

Our dataset comprises 58 targets covering a variety of protein functions: kinases (22), enzymes (12), nuclear receptors (9), proteases (7), G-protein coupled receptors (5), cytochromes (2), and chaperone (1). For details, see Table 2. We have identified a subset of 24 targets whose docking scores are of relatively high quality based on their ability to achieve enrichment of experimental active labels (details are given in Section 3.2.3). These high-quality targets are involved in a range of diseases and are thus considered of great interest in drug discovery (examples can be seen in Table S2 of the SI).

#### 3.2.2 Ligand Selection and Clustering

**Figure 3:**
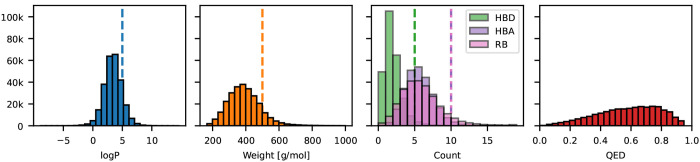
Distribution of molecular properties in the dockstring dataset. Most molecules in our dataset fulfill “Lipinski’s rules of five”^84^ (vertical dashed lines) for the properties depicted (logP, molecular weight, number of hydrogen bond donors [HBD], hydrogen bond acceptors [HBA], and rotatable bonds [RB]). In addition, the QED distribution is left-skewed and peaks at 0.75, further suggesting that most molecules in our dataset are druglike.

ExCAPE is a large database that aggregates results from a variety of assays in PubChem and ChEMBL, many of them from real screening experiments for hit discovery. Furthermore, it sets explicit filters for physicochemical properties such as molecular weight and number of heavy atoms to further promote druglikeness. Indeed, we found that most molecules in our dataset fulfill Lipinski’s rules^84^ and feature favorable QED profiles (Figure 3).

**Figure 4:**
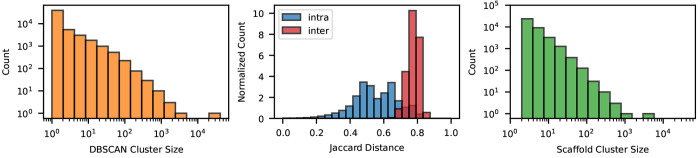
Cluster analysis of dockstring dataset. (Left) Distribution of clusters grouped by the DBSCAN algorithm using the Tanimoto distance. (Middle) Normalized count of Jaccard distances between molecules within the same cluster (blue) and between different ones (red). (Right) Distribution of clusters grouped by scaffold. Here, only molecules from the second and third largest clusters are considered.

We performed cluster analyses with two different techniques: DBSCAN (Density-Based Spatial Clustering of Applications with Noise),^85^ a data-type agnostic clustering algorithm, and Bemis–Murcko scaffold decomposition, which is especially designed for molecules. Given a cluster and a query point, DBSCAN assigns a point to the cluster if it is within the ε-neighborhood of one of its core points (where a core point is one that has a minimum number of neighbors from the same cluster). DBSCAN found 52,000 clusters, where the biggest one covered over 15% of the dataset and 31,000 clusters contained only a single molecule (Figure 4, left). The Jaccard distance within the same cluster was significantly smaller than the distance between different clusters, with little overlap of the two (Figure 4, middle).

Bemis–Murcko decomposition is rooted in the concept of molecular scaffolds.^62^ A scaffold is defined as the union of the ring systems in a molecule plus the linker atoms between them. Thus, there are many possible molecules with the same scaffold that differ only in their side chains and atom types. Molecules with the same scaffold are structurally similar and are expected to have similar properties. We found that our dataset contains 102,000 Bemis–Murcko scaffolds. They showed a similar distribution to DBSCAN clusters, with the most popular scaffold standing out from the rest and 64,000 single-molecule scaffold clusters (Figure 4, right). Overall, these results confirm that molecules in our dataset are diverse.

#### 3.2.3 Docking Scores

We computed docking scores for every target–ligand pair in our dataset, resulting in more than 15 million data points (see Section D of the SI for computational details). To our knowledge, this is the first dataset that computes the full score matrix of a large ligand set against a high number of protein targets, making it ideal for the design of meaningful benchmark tasks in settings such as multiobjective optimization and transfer learning.

**Figure 5:**
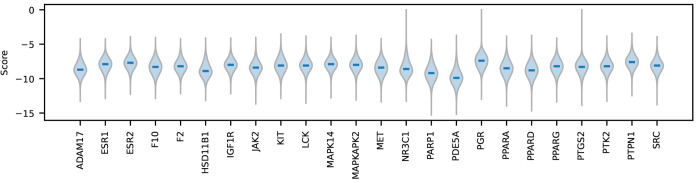
Distribution over docking scores (in kcal/mol) for a subset of high-quality targets in the dockstring dataset in alphabetical order. The tails of each violin plot represent the minimum and maximum docking score for each target. The blue vertical bars indicate the median. For this plot, docking scores greater than zero were set to zero.

**Figure 6:**
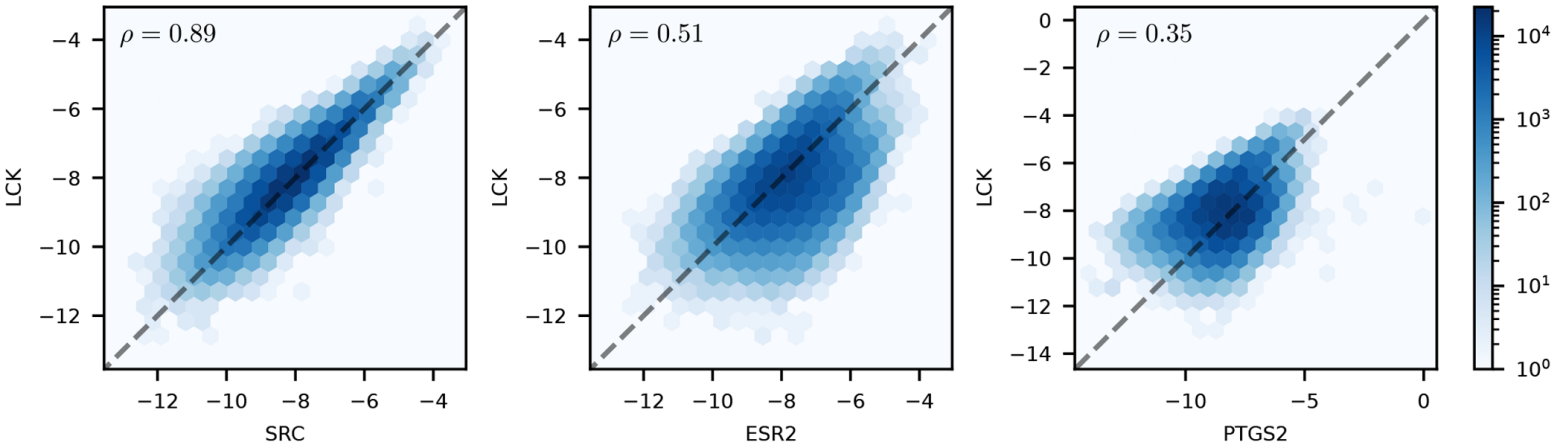
Correlations of docking scores (in kcal/mol) between the kinase LCK and three other targets from the dockstring dataset: SRC, a target from the same family (left), ESR2, a nuclear receptor (middle), and PTGS2, a cyclooxygenase (right). Unlike target independent molecular properties (e.g., logP and QED), docking scores can correlate significantly between targets according to their structural similarity.

We found that the docking scores were similarly distributed for most proteins, ranging between −4 and −13, as can be seen in Figure 5 (note that in the original AutoDock Vina publication^39^ scores are reported in kcal/mol, but for our purposes scores can be treated as a unitless quantity). Docking scores attempt to correlate with binding free energy, so more negative scores suggest stronger binding. We also found that targets that were functionally related or were homologues (i.e., proteins with high sequence similarity such as ESR1 and ESR2) exhibited high correlation, whereas unrelated targets tended to show medium or poor correlation (Figure 6). This supports the claim that dockstring scores are biologically meaningful.

**Figure 7:**
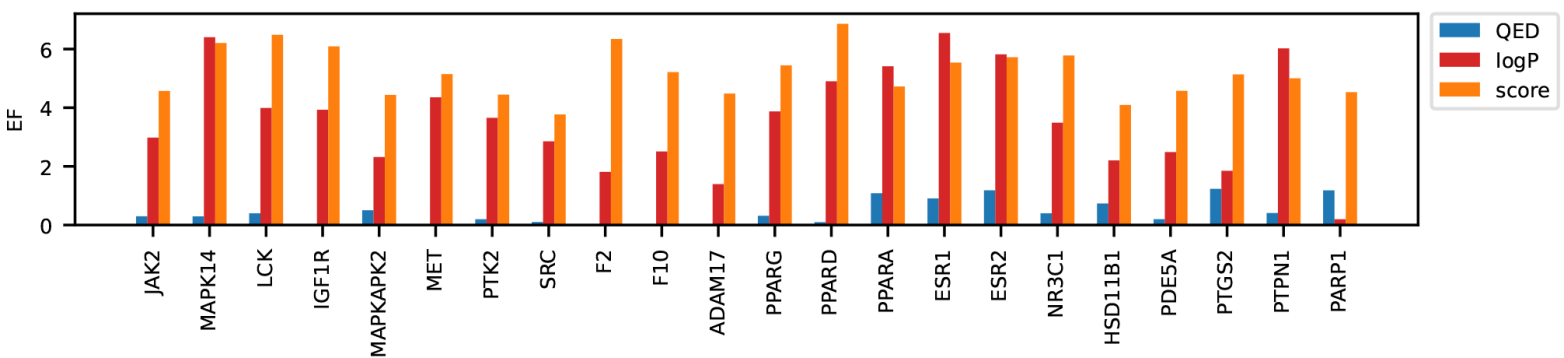
Enrichment factor (EF) of the docking scores (orange) and two target-independent molecular properties, QED (blue) and logP (red), for the high-quality targets in the dockstring dataset in alphabetical order. For most targets, docking scores yielded higher EF than that of the logP or QED.

We assessed the quality of each target’s docking scores based on their enrichment factor (EF), using experimental activity labels from ExCAPE as the reference (Figure 7). Such assessment was necessary because docking is known to perform differently on different proteins, and the optimal docking workflow may vary from one protein to another.^20^ We found that docking scores achieved the highest enrichment overall, although they were surpassed by a small difference by logP in a few targets. This result can be explained because greasy molecules bind nonspecifically to many targets with hydrophobic pockets. However, since this kind of binding is not selective, it may reduce efficacy and increase the risk of toxicity. Therefore, molecules with high logP are usually discarded in drug discovery projects.^86^ Finally, QED achieved very low to no enrichment. Overall, our results indicate that our preparation and docking protocols are effective and yield meaningful docking scores.

#### 3.2.4 Docking Poses

A typical docking simulation results in two outputs: docking poses, which are conformations of the ligand in the binding pocket, and their corresponding docking scores, which predict the strength of the ligand–target interaction. Scores are convenient for ranking compounds in virtual screening workflows. However, they are an approximate heuristic and provide little insight into protein–ligand interactions. By contrast, poses are more interpretable and can help discriminate false positives. Finally, poses can be used as input to ML algorithms that exploit 3D structure information. An example of such models are ML-based scoring functions which produce docking scores from docking poses, which have attracted considerable interest in recent years.^87^ For these reasons, each docking score in our dataset is released together with its corresponding docking pose, adding up to more than 15 million conformations. To our knowledge, the dockstring dataset is the first to include this type of information.

### 3.3 Benchmarks

#### 3.3.1 Regression

A variety of classical regression algorithms were trained on 1024-dimensional binary Morgan fingerprints^88^ with a radius of two: ridge and lasso regression,^89^ gradient-boosted decision trees (XGBoost),^90^ exact GPs,^91^ and sparse GPs.^92^ In addition, two newer algorithms leveraging graph neural networks were also employed, namely, MPNN^93^ and Attentive FP.^94^

The regression performance of the baselines on the five benchmark targets is shown in Table 3. Performances on predicting logP and QED are also shown to help gauge the relative difficulty of the proposed tasks. First, note that classical methods are handily outperformed by deep learning methods. The worst ranking methods are ridge and lasso regression, which are linear models and yield coefficients of determination *R*^2^ ranging between 0.242 and 0.706. In contrast, the best ranking model is Attentive FP, a graph deep neural network, with coefficients ranging between 0.627 and 0.910 and beating every other method by a significant margin. Second, note that some targets seem to be more difficult than others. The easiest target is PARP1, whereas the most challenging target is PGR. This is in contrast with logP and QED, where the graph ML methods achieve perfect or near-perfect performance. This strongly supports the use of docking scores instead of logP and QED to benchmark high-performing models.

**Table 3:** Regression Performance for Select Tasks^a^

Target	Ridge	Lasso	XGBoost	GP (exact)	GP (sparse)	MPNN	Attentive FP
logP	0.640	0.640	0.734	0.707	0.716	0.953	**1.000**
QED	0.519	0.483	0.660	0.640	0.598	0.901	**0.981**
ESR2	0.421	0.416	0.497	0.441	0.508	0.506	**0.627**
F2	0.672	0.663	0.688	0.705	0.744	0.798	**0.880**
KIT	0.604	0.594	0.674	0.637	0.684	0.755	**0.806**
PARP1	0.706	0.700	0.723	0.743	0.772	0.815	**0.910**
PGR	0.242	0.245	0.345	0.291	0.387	0.324	**0.678**
Average rank	6.04	6.96	4.17	4.71	2.88	2.25	**1.00**

a Full results are in Tables S3 and S4. Numbers represent the mean coefficient of determination (*R*^2^ score) averaged over three runs (highest is better). The best score in each row is in **bold**. The average rank includes only the dockstring targets (excluding logP and QED).

Regression performance was also evaluated as mean squared error (MSE, Table S5) and mean absolute error (MAE, Table S6), obtaining results very similar to those of *R*^2^.

#### 3.3.2 Virtual Screening

The goal of virtual screening is to identify actives from a large library that is too big to be analyzed experimentally. Our screening benchmark evaluated the ability of ML models to select a subset of molecules from the ZINC20 database with docking scores better (i.e., lower) than a threshold (the 0.1 percentile). We trained models on the docking scores of PARP1, KIT, and PGR and used these models to rank all molecules in ZINC20 according to their predicted docking score. Then, we selected the top 5000 predicted compounds and computed their actual docking scores with dockstring. Finally, molecules with a docking score lower than the 0.1 percentile were labeled as active, and the EF of the top 5000 selected with respect to ZINC20 was calculated.

We selected the methods attentive FP and ridge regression from Section 3.3.1 as baselines for virtual screening. The former was chosen for its high regression scores, while the latter was selected based on its simplicity and low computational cost. Implementation details were the same as in Section 3.3.1. We also included fingerprint similarity search (FSS, fingerprint nearest neighbor) for comparison with a more classical virtual screening method. The implementation of this method is described in Section 2.4.

In general, the FSS baseline was the poorest of the three, yielding the lowest EF in KIT and PARP1 and just slightly better EF than ridge regression in PGR (Table 4). In contrast, Attentive FP was clearly superior to other methods in all targets, mirroring its positive performance in the regression baseline. Regarding target difficulty, the easiest protein for screening seemed to be KIT, thus disagreeing with previous regression results, where PARP1 seemed to be the easiest target. This suggests that in-distribution prediction difficulty (i.e., regression with a test set from the same dataset as the training set) may be different than out-of-distribution prediction difficulty (i.e., virtual screening of a library different from the training set) in some targets. Therefore, property oracles such as the docking engine in dockstring, which allow instantaneous labeling of external datasets and libraries on the fly, may be necessary to evaluate out-of-distribution prediction and to perform realistic prospective validation.

**Table 4:** Enrichment Factors (EF) for Virtual Screening Tasks^a^

Target	Threshold score	FSS	Ridge	Attentive FP
KIT	–10.7	239.2	451.6	**766.5**
PARP1	–12.1	313.1	325.9	**472.2**
PGR	–10.1	161.4	120.5	**461.3**

a Higher is better. For each target, a threshold score is given below which a ligand is considered active. Highest possible EF for the chosen thresholds is 1000.

#### 3.3.3 *De Novo* Molecular Design

**Figure 8:**
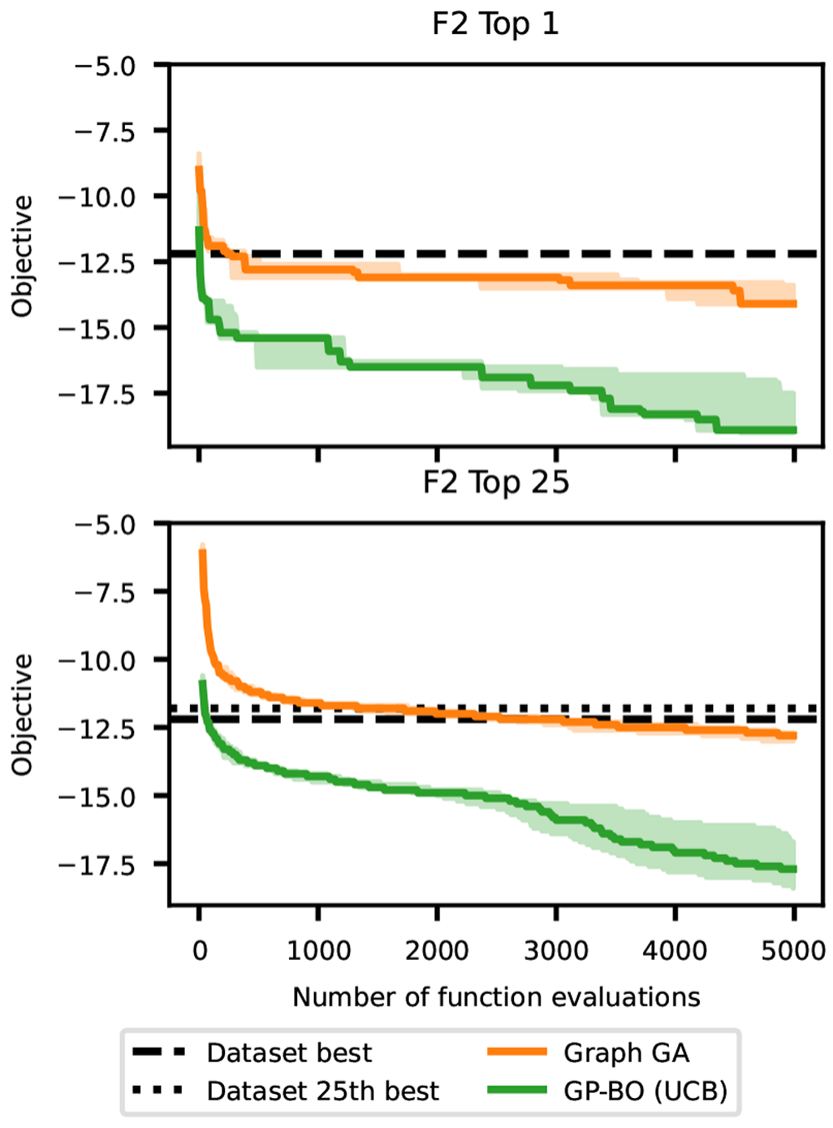
Results for baseline algorithms on the F2 *de novo* molecular design task *without* the QED penalty. The objective value of the best molecule found so far is shown as a function of the number of objective function calls. The solid lines indicate the median and the shaded area the minimum and maximum over three runs. The black dashed line indicates the best value in the dockstring dataset.

**Figure 9:**
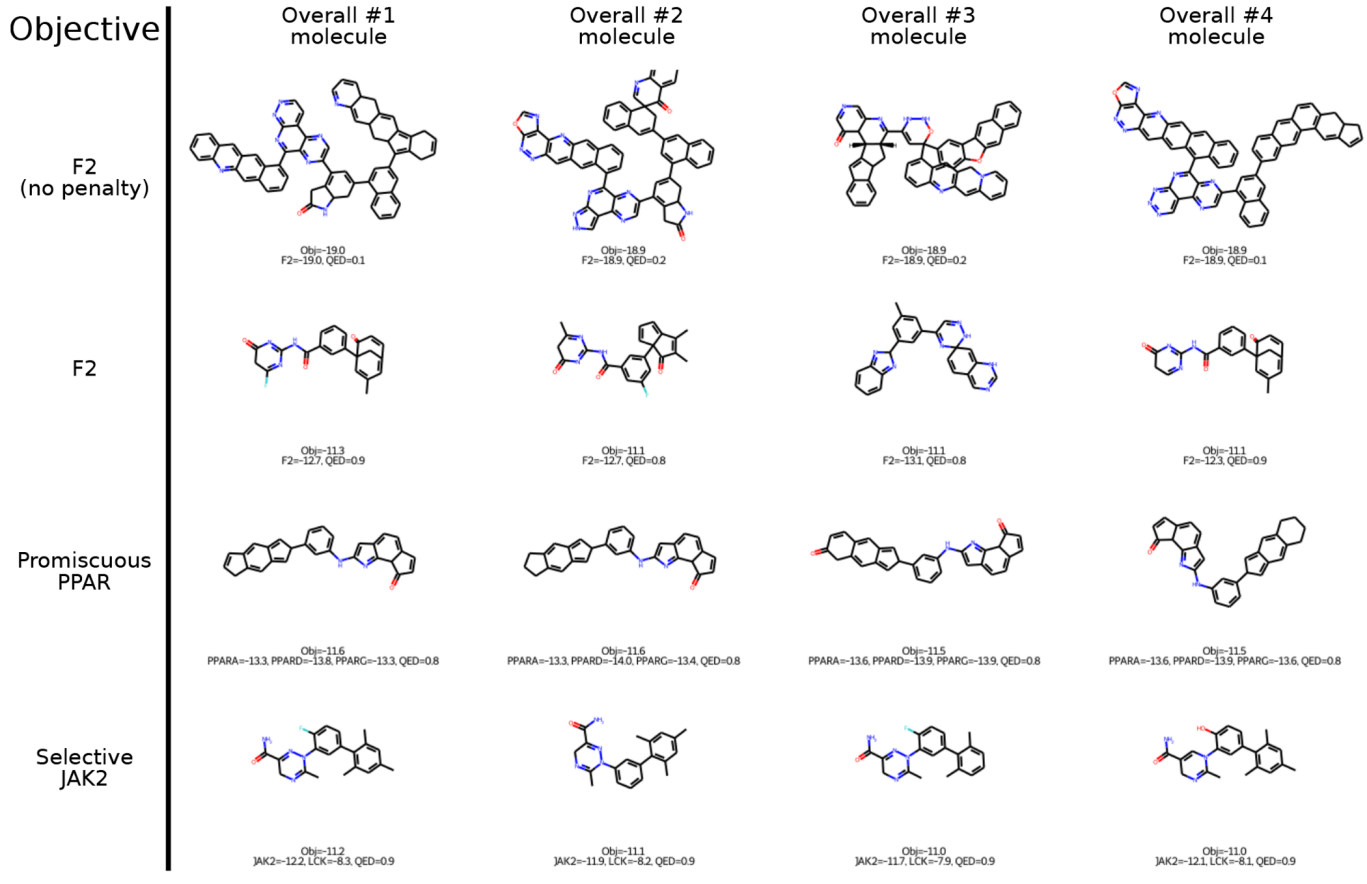
Top four molecules for F2 (no penalty), F2, Promiscuous PPAR, and Selective JAK2.

With our novel *de novo* design tasks, we compared two genetic algorithms (GAs), a simple GA based on SELFIES^80^ and the graph GA by Jensen,^95^ with Gaussian process Bayesian optimization (GP-BO) approaches using the upper confidence bound (UCB) and expected improvement (EI) acquisition functions (for details, see Section 2.4). We also included a random baseline which randomly selected molecules from the ZINC20 dataset.

dockstring introduces three *de novo* benchmark tasks: optimization of F2 docking scores (F2), joint optimization of PPAR nuclear receptors (Promiscuous PPAR), and adversarial optimization of JAK2 against LCK (Selective JAK2). Initially, we defined naive versions of these tasks that did not include a penalty to enforce druglikeness. These unpenalized tasks were solved easily, with most methods quickly finding molecules with better objective values than the best in the training set in just tens of iterations (Figure 8). However, although the molecules produced were better in terms of the objective function alone, they were large, lipophilic, and highly undruglike as per Lipinski rules and QED (Figure 9, first row), which would make them unsuitable for therapeutic applications. Such preference for undesirable molecules may be explained by the inherent biases of docking algorithms. On the one hand, docking tends to give high scores to large molecules, since they can potentially establish more interactions with the target and most scoring functions are additive. Therefore, large molecules with high docking scores are often false positives.^68^ On the other hand, hydrophobic molecules bind nonspecifically to many proteins with hydrophobic regions in their binding pockets, which can lead to off-target effects, toxicity, and decreased efficiency. Therefore, highly hydrophobic molecules are also undesirable.^69^

To make the tasks more challenging and enforce druglikeness explicitly, we added a QED penalty to each of the naive tasks. The functional form chosen was . Since QED ranges between 0 and 1, this penalty will be 0 at minimum and 10 at maximum, which covers approximately the same numeric value of docking scores. The full objective functions can be found in Section 2.3.3.

**Figure 10:**
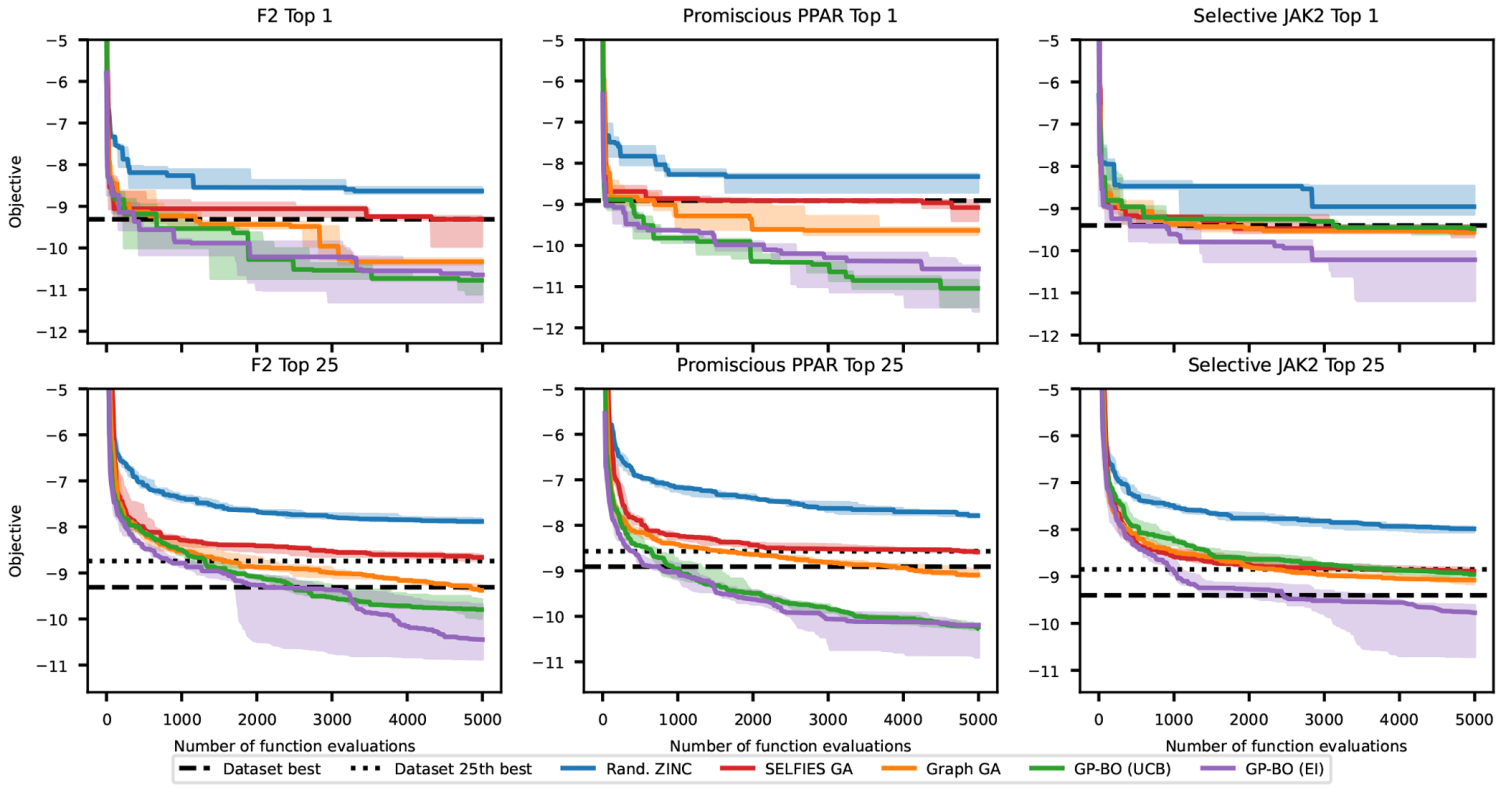
Results for baseline algorithms on three different *de novo* molecular design tasks. The objective values of the first and 25th best molecule found so far are shown as a function of the number of objective function calls. The solid lines indicate the median and the shaded area the minimum and maximum over three runs. The black dashed line indicates the best (and 25th best) value in the dockstring dataset.

The optimization trajectories of the penalized tasks (Figure 10, top) were generally flatter than that of the naive unpenalized one, suggesting that they are more difficult. In F2, three of the methods beat the best molecules in the dataset by a large margin, compared with two methods for Promiscuous PPAR and just one method for Selective JAK2, suggesting that the task difficulty increases in that order. In general the GP-BO algorithms tend to significantly outperform the GAs, although GP-BO with UCB acquisition is comparable to the GAs for the selective JAK2 task. Random sampling of ZINC molecules yielded the worst performance, which is expected since this strategy does not learn from past molecules unlike other optimization methods. The objective value of the 25th best molecule so far (Figure 10, bottom) showed a similar relative performance of optimization algorithms as in the single best molecule, except that differences between algorithms were more pronounced. In addition, only a single method, GP-BO with EI acquisition, was able to find 25th best molecules better than the training set in all tasks. This suggests that finding multiple high-performing molecules is more challenging than finding a single high-performing molecule, as expected.

Molecules generated in F2 and Promiscious PPAR featured conjugated ring structures which are relatively unusual in successful drugs (Figure 9, second and third rows). Selective JAK2 yielded smaller molecules, with interesting structures, druglike appearance, and higher QED values, although all the top molecules shared a similar backbone (Figure 9, fourth row). We hypothesize that adversarial objectives based on correlated docking scores may be an effective way to avoid docking biases compared to simple penalties based on QED, since exploiting the bias of docking scores for high molecular size and lipophilicity may benefit one component of the objective while hurting another. Future work is needed to further study and verify this effect.

**Figure 11:**
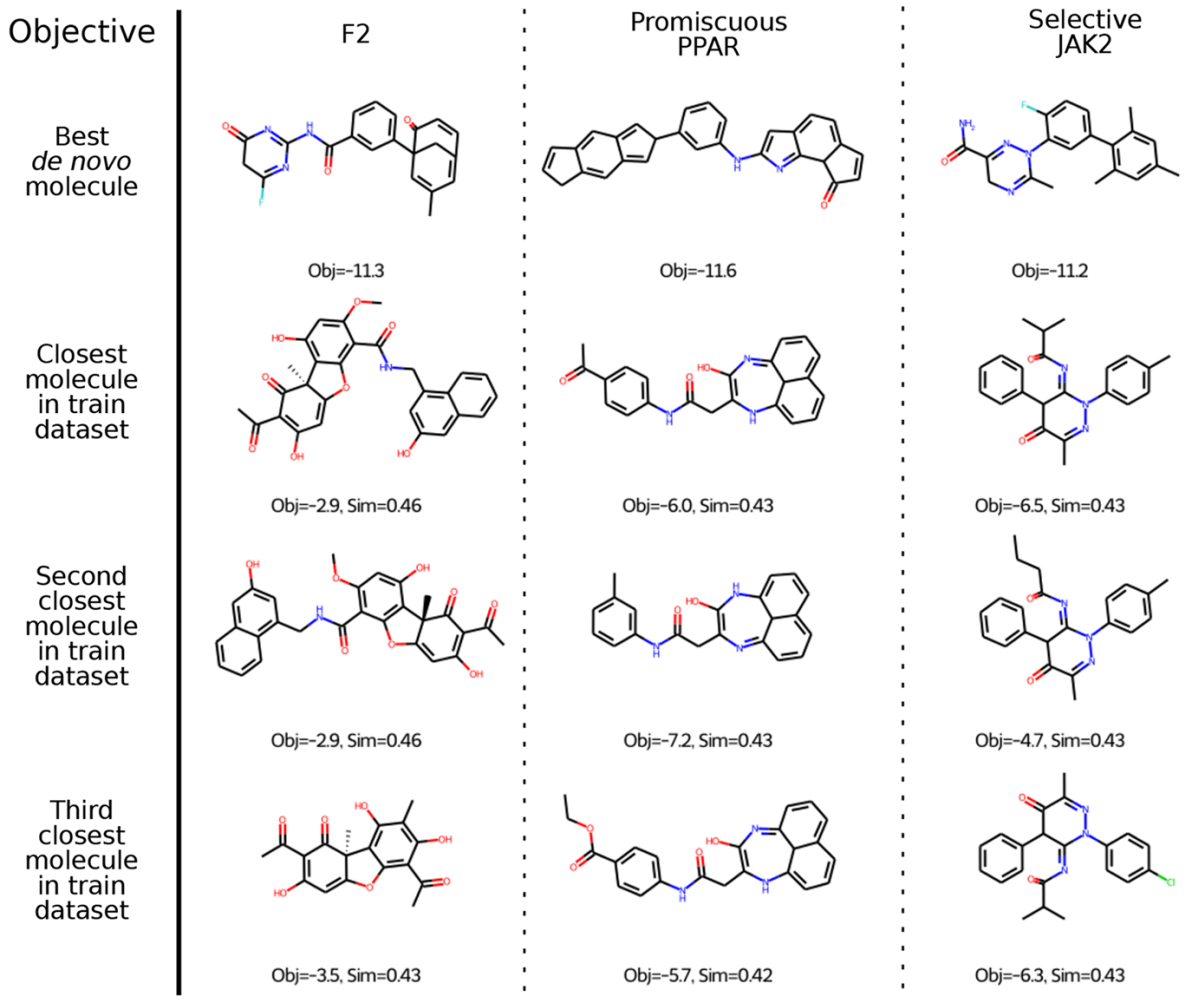
Most similar molecules in training set for the three objectives F2, Promiscuous PPAR, and Selective JAK2.

In general, the best molecules in the three tasks are unique and distinct from the training set (Figure 11). For F2 and Promiscuous PPAR, none of the top molecules has a generic Murcko scaffold in the training set. For Selective JAK2, all of the top 12 molecules share a generic Murcko scaffold with a training set molecule, but the most similar molecule is still reasonably different.

To compare the difficulty of our *de novo* design tasks with other popular benchmark functions, we assessed the performance of two baseline models when optimizing logP and QED. Our results suggest that neither logP nor QED are appropriate objectives for model evaluation. LogP was remarkably easy to optimize for all methods, in line with previous regression results indicating that this property is not challenging enough (cf. Table 3). Furthermore, it promoted molecules that were highly unrealistic and not druglike (Figure S1 of the SI). On the other hand, QED seemed to be maximized by molecules already in the dataset, and it could not be improved further than 0.948. Since QED is itself a scalarized multiobjective function of several physicochemical properties, this suggests that many existing molecules in chemical depositories are already in the QED Pareto frontier. Therefore, QED may be more useful as a soft constraint for druglikeness (as employed in this work) than as a benchmark objective.

#### 3.3.4 Pose Analysis of *De Novo* Kinase Inhibitors

Protein kinases are relevant pharmaceutical targets because of their important role in cell signaling and cancer. They activate and inactivate other proteins via phosphorylation of the hydroxyl group of a serine, threonine, or tyrosine residue. Even though many kinases are structurally similar, each kinase is tightly regulated, and they achieve high specificity in their respective signaling pathways.^96^ However, from a medicinal standpoint, their structural similarity makes it challenging to design selective inhibitors.

Our *de novo* design task Selective JAK2 has the goal to propose molecules which bind strongly to the tyrosine kinase JAK2 but only bind weakly to the related tyrosine kinase LCK. Here, binding is determined by Autodock Vina docking scores. As discussed in the previous section, we found that this task was the hardest for our baseline algorithms, which could be explained by the high correlation between JAK2 and LCK scores (0.8). Still, the baseline GP-BO with acquisition function EI achieved a considerable improvement over the best molecule in the training set. In order to understand the binding mode of the top *de novo* molecule proposed, we analyzed its docking poses in JAK2 and LCK and compared it to previously known inhibitors.

All human kinase proteins possess a kinase domain that catalyzes the transfer of a phosphate group from ATP onto the substrate. Although diverse in sequence, the kinase catalytic domain is remarkably similar in its 3D structure across different kinase proteins.^97^ It consists of a C-terminal lobe rich in α-helices, an N-terminal lobe rich in β-sheets, and a flexible loop, called the hinge, which connects the two lobes. ATP binds between the two lobes, establishing hydrogen bonds between its adenine moiety and the hinge region. Similar bonds are also formed by many kinase inhibitors, thus competing directly with ATP. However, because the ATP-binding site is highly conserved, achieving selectivity through interactions with the hinge region is hard.^97,98^

**Figure 12:**
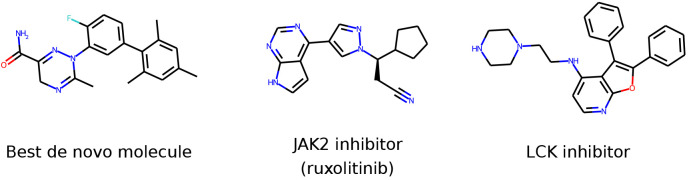
Structure of the best *de novo* molecule in Selective JAK2, an example JAK2 inhibitor (cancer drug ruxolitinib) and an example LCK inhibitor.

**Figure 13:**
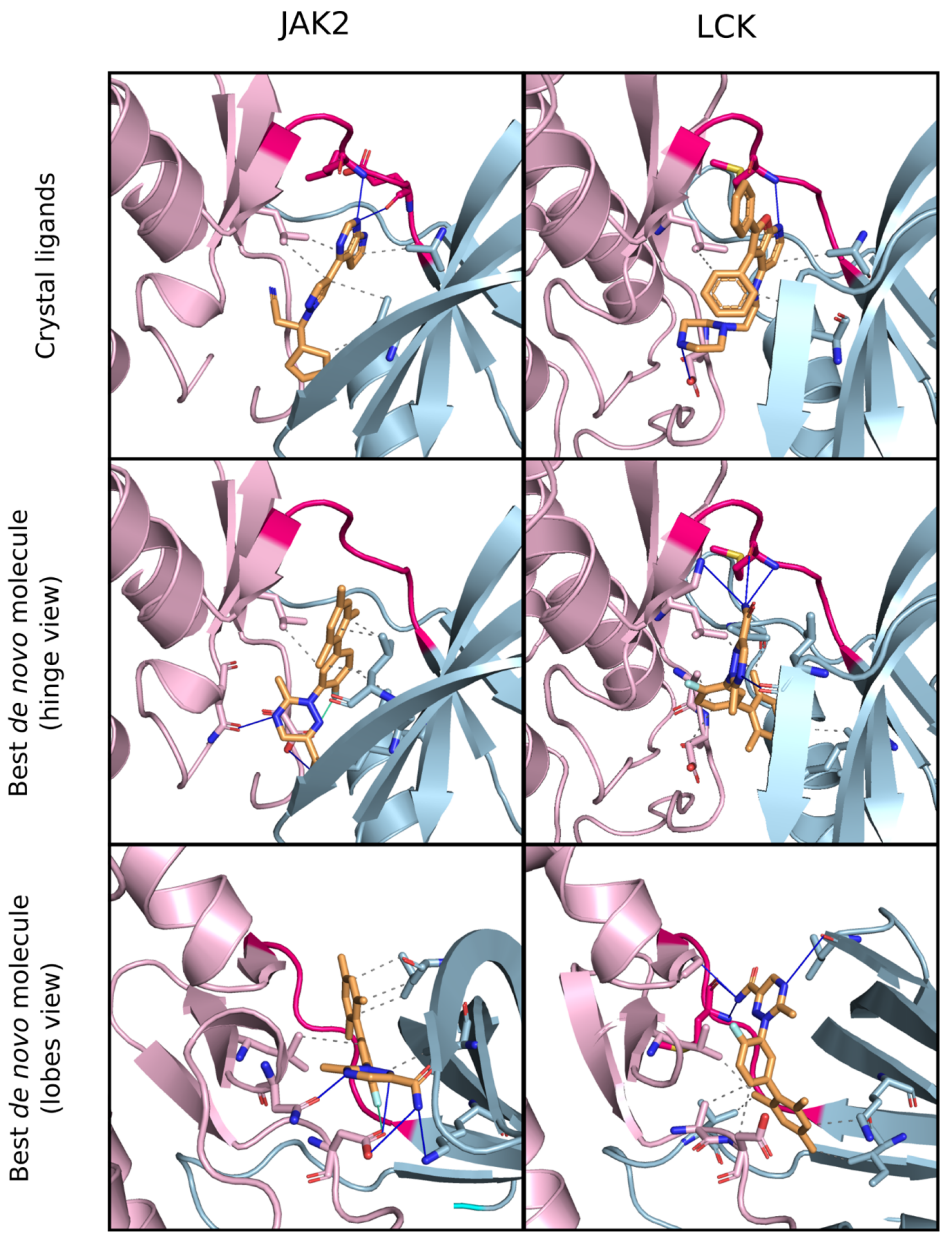
Pose analysis of the top-scoring *de novo* molecule in the Selective JAK2 task and comparison with two known inhibitors. Known inhibitors in crystal structures of JAK2 (top, left) and LCK (top, right) form hydrogen bonds with the hinge that connects the N- and C-terminal lobes. This interaction is typical of type I kinase inhibitors. According to Autodock Vina, the top molecule features different binding modes in JAK2 (middle and bottom, left) and LCK (middle and bottom, right). In JAK2, it does not interact with the hinge, but rather it forms bonds with distant residues of the N- and C-terminal lobes. Inhibitors that bind the kinase at different sites simultaneously are called type V inhibitors. In LCK, the top molecule behaves similarly to most kinase inhibitors, forming hydrogen bonds with the hinge. (Figures produced with PyMOL^49^ and interactions highlighted with PLIP.^99^).

We analyzed the poses of the top *de novo* molecule against JAK2 and LCK using PLIP, a 3D interaction profiler.^99^ For comparison, we also analyzed the interactions of two example inhibitors (shown in Figure 12) for which crystal structures were known. The first inhibitor, ruxolitinib, is a JAK2-targeting drug approved to treat myelofibrosis, a rare type of bone marrow cancer. Applying PLIP to the crystal structure (PDB 6VGL(100)) showed that ruxolitinib interacted with the hinge in a traditional fashion by establishing hydrogen bonds with its backbone chain (Figure 13). Inhibitors with this binding profile are usually referred to as type I kinase inhibitors.^101^ In contrast, the top *de novo* molecule did not appear to interact with the hinge according to Autodock Vina. Rather, it formed hydrogen bonds and hydrophobic interactions with distant regions of the N- and C-terminal lobes. Inhibitors of this type, which bind a kinase simultaneously at different sites, are known as type V inhibitors.^101^ They have been described in the literature as potential routes to achieving selectivity.^102^ Notably, the top *de novo* molecule also established a halogen bond with an aspartate residue of the C lobe. Halogen bonds are enthalpically favorable and can be exploited to enhance binding affinity and specificity.^103^ Interestingly, among the 20 molecules in the training set with the highest score for Selective JAK2, there were only four molecules that presented halogen groups, and none of them had the same scaffold as the top *de novo* molecule (Figure S2).

PLIP analysis of LCK poses suggested a different binding mode of the top *de novo* molecule in LCK and JAK2. Both the example LCK inhibitor (PDB 2OF2(104)) and the top molecule appeared to interact with the LCK hinge through hydrogen bonds, similar to how ruxolitinib interacted with JAK2. However, in JAK2, the top molecule exhibited no interactions with the hinge, as previously described. Therefore, the Vina poses suggest that the top *de novo* molecule could achieve selectivity through a dual binding mode mechanism. In JAK2, it may adopt an “amide-out” configuration, with the amide group, heterocyclic nitrogens, and fluorine atom establishing enthalpically favorable bonds with the N- and C-terminal lobes. In LCK, it may adopt an “amide-in” configuration, with the amide group forming hydrogen bonds with the hinge. Interestingly, in the LCK “amide-in” configuration, the heterocycle was aligned along a different plane than corresponding rings in the example inhibitors, which may also account for the lower Vina score.

To determine whether the poses predicted by Vina were robust, we examined other poses of the top *de novo* molecule against JAK2 and LCK. We found that the second-best pose of the top molecule pointed to the same binding mode, with RMSD values of 0.18 Å in JAK2 and 0.77 Å in LCK. We also examined the second-best *de novo* molecule and found identical binding modes in both JAK2 and LCK (Figure S3). Still, it is worth emphasizing that our binding analysis and, more generally, the Selective JAK2 task depend directly on Autodock Vina scores and poses. Further experimental verification would be needed to confirm that the dual binding mode of the top *de novo* molecule with respect to JAK2 and LCK is not a docking artifact. Therefore, *de novo* design tasks in dockstring should not be regarded as a substitute to rational drug design and experimental validation. Instead, they should be viewed as sophisticated benchmarks for ML models that require deep understanding of the underlying chemistry in order to be solved.

## 4 Conclusions and Outlook

With the release of dockstring, we hope to make docking-based benchmarking as accessible as possible and thus enable the scientific community to benchmark algorithms against challenging and relevant tasks in drug discovery. The simple and robust Python package enables automatic computation of docking scores and poses, facilitating the acquisition of new labels and the design of sophisticated workflows of virtual screening or molecular optimization—even by researchers with little domain expertise. The dataset of unprecedented size and diversity allows users to train models without having to spend significant computational resources. Furthermore, it provides curated and standardized training and test sets for each benchmark so that models are compared fairly. This consideration is particularly important to ML for chemistry, given that different dataset splits can lead to largely disparate results due to the biased and undersampled nature of chemical space. Our training and test sets were constructed with cluster splitting to minimize the chances of overfitting and data leakage. Finally, the set of benchmark tasks is carefully designed so that they are relevant to both the ML and the drug discovery communities, covering a variety of ML settings and biological problems.

The possibilities for tasks based on docking are by no means exhausted in this paper, and we plan to continue improving the package, dataset, and benchmarks (see Section E of the SI for our maintenance plan). The following areas are of particular interest. First, there is room to adapt and improve the *de novo* design tasks, in particular, the objective functions, to encourage the generation of molecules with better pharmacokinetic properties and more feasible synthetic pathways. Second, the range of protein targets included in dockstring makes it well suited to multiobjective tasks such as transfer learning, self-supervised learning, and few-shot learning. These are left for future work. Third, docking scores are considered a relatively limited predictor of bioactivity, because, among other reasons, they use a static binding site and force fields which are poorly calibrated for certain metal ions, for instance. Therefore, drug discovery projects tend to employ more expensive computational techniques and experimental assays in later stages of the drug discovery pipeline. Developing transfer learning and multifidelity optimization tasks for different predictors of activity on the same dockstring target would be a relevant avenue of future research.

## Data and Software Availability

The dockstring molecular docking package, the dockstring dataset, and code for the baselines are available at https://dockstring.github.io. All components are released under the Apache 2.0 license.

## Supporting Information Available

Section A: Popular molecular benchmarks and their weaknesses. Section B: Some medically relevant proteins in dockstring and their biological significance. Section C: Additional information about the baseline experiments, including implementation details of the models and additional results and visualizations. Section D: Computational details about the creation of the dataset and the execution of the baseline experiments, including average docking run times for each protein target in dockstring. Section E: Maintenance plan for our package and dataset. (PDF)
