# Supplementary material for: DOCKSTRING: Easy Molecular Docking Yields Better Benchmarks for Ligand Design
Source: J Chem Inf Model. 2022 Jul 18;62(15):3486–502. doi: 10.1021/acs.jcim.1c01334 (PMC9364321; doi:10.1021/acs.jcim.1c01334)
Supplement: Supplementary file 1 — ci1c01334_si_001.pdf [file ci1c01334_si_001.pdf]

Supporting Information

DOCKSTRING: Easy Molecular Docking Yields Better Benchmarks for Ligand Design

Miguel García-Ortegón,[*,†] Gregor N. C. Simm,[‡] Austin J. Tripp,[‡] José Miguel Hernández-Lobato,[‡] Andreas Bender,[¶] and Sergio Bacallado[*,†]

†*Statistical Laboratory, University of Cambridge, Centre for Mathematical Sciences, Wilberforce Rd, Cambridge CB3 0WB, United Kingdom*

‡*Department of Engineering, University of Cambridge, Trumpington St, Cambridge CB2 1PZ, United Kingdom*

¶*Yusuf Hamied Department of Chemistry, University of Cambridge, Lensfield Rd, Cambridge CB2 1EW, United Kingdom*

E-mail: mg770@cam.ac.uk; sb2116@cam.ac.uk

A Popular Molecular Benchmarks and Their Relevance to Drug Discovery

Table S1: Popular molecular benchmarks and their relevance to drug discovery.

	Description	Relevance for drug discovery
logP	Ratio of the concentrations of a compound in a mixture of an organic solvent and water.	Some heuristic rules consider the logP (e.g., less than five in Lipinski's rule of five[1]) since molecules with a high logP often suffer from unspecific binding and safety liabilities.
quantitative estimate of drug-likeness (QED)	The **Q**uantitative **E**stimate of **D**ruglikeness measures similarity to marketed drugs based on simple physicochemical properties.	QED has limited predictive power to discriminate approved drugs from decoys.[2] Further, it is not selective against a particular disease or target protein.
SAS	Based on the similarity to synthesizable compounds, the **S**ynthetic **A**ccessibility **S**core estimates how difficult it is to synthesize a molecule.	Synthesizability is a pre-requisite for any molecule to be assayed *in vitro* or *in vivo*, but it does not inform about efficacy or safety.
Molecular mass	Often referred to as the molecular weight.	While some heuristic rules for drug-likeness consider molecular weight (e.g., less than 500 Da in Lipinski's rule of five), it offers little information about efficacy and safety.
Docking scores	Prediction of binding free energy between a molecule (ligand) and a protein (target).	Popular method in virtual screening with the goal to enrich a subset with bioactive compounds from an extensive molecular library.

B DOCKSTRING Target Proteins

Table S2: Examples of high-quality targets in the DOCKSTRING dataset that are relevant for drug discovery. Drugs in this table are all small molecules.[3]

Target	Group	Biological significance	Drug
Janus kinase 2 (JAK2)	Kinase	Cell signaling via the JAK-STAT pathway. Misregulated or mutated in a range of cancers.	Ruxolitinib. Selective JAK inhibitor used to treat myelofibrosis, a rare type of bone marrow blood cancer.
Tyrosine-protein kinase (KIT)	Kinase	Cell-surface receptor and signal transducer for cytokines. Misregulated or mutated in a range of cancers.	Axitinib. Used to treat renal cell carcinoma.
Hepatocyte growth factor receptor (MET)	Kinase	Cell-surface receptor and signal transducer for the hepatocyte growth factor. Initiator of the MET signaling pathway. Misregulated or mutated in a range of cancers.	Crizotinib. Used for the treatment of non-small cell lung cancer (NSCLC).
Thrombin (F2)	Protease	Catalizes the cleavage of soluble fibrinogen into insoluble fibrin to promote blood coagulation during blood clotting.	Bivalirudin. Anticoagulant used to prevent thrombosis in patients under heparin treatment.
Peroxisome proliferator-activated receptor alpha (PPARA)	Nuclear receptor	Regulator of liver metabolism. Activates uptake and utilization of fatty acids.	Clofibrate. Used to control high cholesterol and triglyceride levels in the blood.
Cyclic guanosine monophosphate specific phosphodiesterase type 5 (PDE5A)	Enzyme	Degrades the messenger cGMP, promoting vasodilation and increased blood flow.	Sildenafil (viagra). Used to treat erectile dysfunction.

C Benchmark Details

Code for all baselines is provided at `https://dockstring.github.io`.

C.1 Regression

C.1.1 Additional Details

Clipping positive scores. Docking scores are clipped to a maximum value of +5 before fitting the regression model because there were a small number of huge positive scores (e.g.,+100), which we worried would have a large negative impact on the training of some models. Positive docking scores represent poor binding and therefore predicting the exact value of a positive docking score is uninteresting. For most targets, there were no positive scores and therefore this clipping had no effect.

Train/test split. Specifically, the train/test sets were produced by sorting the clusters by size, then adding all the molecules in the largest cluster to the training set. This process was repeated using the remaining clusters until the 85% of the dataset had been added to the training set. The remaining data points were used as the test set, and consist mostly of small, isolated clusters.

C.1.2 Results

R^2 score. There are several similar definitions of R^2 score. The one used here is the implementation from `scikit-learn` in `https://scikit-learn.org/stable/modules/generated/sklearn.metrics.r2_score.html`. With this definition, perfect prediction gets a score of 1.0, while predicting the dataset mean will have a score of 0.0.

Table S3: Coefficient of determination (R^2, higher is better) for regression baseline methods. Results shown here are the mean of three runs; the full table with standard deviations can be found in the Appendix (Table S4). All standard deviations were small. The vertical line separates classical fingerprint-based methods from deep learning methods. The best score in each row is in **bold**.

Target	Ridge	Lasso	XGBoost	GP (exact)	GP (sparse)	MPNN	Attentive FP
logP	0.640	0.640	0.734	0.707	0.716	0.953	**1.000**
QED	0.519	0.483	0.660	0.640	0.598	0.901	**0.981**
ADAM17	0.597	0.591	0.661	0.638	0.685	0.748	**0.822**
ESR1	0.499	0.478	0.567	0.527	0.584	0.609	**0.729**
ESR2	0.421	0.416	0.497	0.441	0.508	0.506	**0.627**
F10	0.655	0.652	0.685	0.687	0.727	0.743	**0.856**
F2	0.672	0.663	0.688	0.705	0.744	0.798	**0.880**
HSD11B1	0.425	0.424	0.577	0.542	0.612	0.620	**0.793**
IGF1R	0.583	0.574	0.632	0.615	0.666	0.685	**0.799**
JAK2	0.617	0.617	0.686	0.667	0.712	0.759	**0.853**
KIT	0.604	0.594	0.674	0.637	0.684	0.755	**0.806**
LCK	0.670	0.666	0.706	0.708	0.746	0.789	**0.890**
MAPK14	0.592	0.587	0.655	0.640	0.689	0.731	**0.814**
MAPKAPK2	0.662	0.660	0.704	0.699	0.736	0.788	**0.855**
MET	0.587	0.584	0.663	0.633	0.683	0.766	**0.804**
NR3C1	0.258	0.257	0.495	0.439	0.520	0.425	**0.753**
PARP1	0.706	0.700	0.723	0.743	0.772	0.815	**0.910**
PDE5A	0.601	0.600	0.661	0.663	0.702	0.769	**0.851**
PGR	0.242	0.245	0.345	0.291	0.387	0.324	**0.678**
PPARA	0.577	0.575	0.645	0.626	0.675	0.736	**0.823**
PPARD	0.630	0.627	0.686	0.667	0.714	0.782	**0.851**
PPARG	0.641	0.634	0.677	0.662	0.707	0.770	**0.818**
PTGS2	0.322	0.310	0.398	0.349	0.419	0.427	**0.588**
PTK2	0.611	0.603	0.675	0.657	0.700	0.751	**0.839**
PTPN1	0.600	0.596	0.660	0.630	0.677	0.706	**0.790**
SRC	0.663	0.660	0.689	0.692	0.735	0.802	**0.875**
**Average Rank**	6.042	6.958	4.167	4.708	2.875	2.250	**1.000**

Table S4: Full regression results (complete version of Table S3 with standard deviations).

Target	Ridge mean	Ridge std	Lasso mean	Lasso std	XGBoost mean	XGBoost std	GP (exact) mean	GP (exact) std	GP (sparse) mean	GP (sparse) std	MPNN mean	MPNN std	Attentive FP mean	Attentive FP std
logP	0.640	0.000	0.640	0.000	0.734	0.000	0.707	0.003	0.716	0.007	0.953	0.007	**1.000**	0.000
QED	0.519	0.000	0.483	0.008	0.660	0.000	0.640	0.003	0.598	0.053	0.901	0.006	**0.981**	0.001
ADAM17	0.597	0.000	0.591	0.009	0.661	0.000	0.638	0.003	0.685	0.001	0.748	0.031	**0.822**	0.006
ESR1	0.499	0.000	0.478	0.016	0.567	0.000	0.527	0.002	0.584	0.001	0.609	0.016	**0.729**	0.002
ESR2	0.421	0.001	0.416	0.008	0.497	0.000	0.441	0.002	0.508	0.000	0.506	0.001	**0.627**	0.010
F10	0.655	0.000	0.652	0.002	0.685	0.000	0.687	0.001	0.727	0.000	0.743	0.028	**0.856**	0.001
F2	0.672	0.000	0.663	0.009	0.688	0.000	0.705	0.002	0.744	0.000	0.798	0.005	**0.880**	0.001
HSD11B1	0.425	0.000	0.424	0.001	0.577	0.000	0.542	0.003	0.612	0.002	0.620	0.023	**0.793**	0.006
IGF1R	0.583	0.000	0.574	0.010	0.632	0.000	0.615	0.002	0.666	0.001	0.685	0.037	**0.799**	0.003
JAK2	0.617	0.000	0.617	0.000	0.686	0.000	0.667	0.001	0.712	0.002	0.759	0.006	**0.853**	0.003
KIT	0.604	0.000	0.594	0.011	0.674	0.000	0.637	0.002	0.684	0.001	0.755	0.005	**0.806**	0.008
LCK	0.670	0.000	0.666	0.002	0.706	0.000	0.708	0.001	0.746	0.000	0.789	0.026	**0.890**	0.000
MAPK14	0.592	0.000	0.587	0.006	0.655	0.000	0.640	0.001	0.689	0.001	0.731	0.010	**0.814**	0.004
MAPKAPK2	0.662	0.000	0.660	0.001	0.704	0.000	0.699	0.002	0.736	0.001	0.788	0.031	**0.855**	0.010
MET	0.587	0.000	0.584	0.004	0.663	0.000	0.633	0.004	0.683	0.001	0.766	0.011	**0.804**	0.009
NR3C1	0.258	0.002	0.257	0.001	0.495	0.000	0.439	0.003	0.520	0.002	0.425	0.030	**0.753**	0.005
PARP1	0.706	0.000	0.700	0.004	0.723	0.000	0.743	0.002	0.772	0.002	0.815	0.010	**0.910**	0.002
PDE5A	0.601	0.000	0.600	0.000	0.661	0.000	0.663	0.002	0.702	0.004	0.769	0.013	**0.851**	0.003
PGR	0.242	0.002	0.245	0.001	0.345	0.000	0.291	0.007	0.387	0.000	0.324	0.096	**0.678**	0.008
PPARA	0.577	0.000	0.575	0.002	0.645	0.000	0.626	0.002	0.675	0.001	0.736	0.017	**0.823**	0.008
PPARD	0.630	0.000	0.627	0.001	0.686	0.000	0.667	0.003	0.714	0.001	0.782	0.010	**0.851**	0.003
PPARG	0.641	0.000	0.634	0.007	0.677	0.000	0.662	0.002	0.707	0.001	0.770	0.010	**0.818**	0.003
PTGS2	0.322	0.001	0.310	0.017	0.398	0.000	0.349	0.004	0.419	0.003	0.427	0.004	**0.588**	0.020
PTK2	0.611	0.000	0.603	0.003	0.675	0.000	0.657	0.003	0.700	0.002	0.751	0.013	**0.839**	0.002
PTPN1	0.600	0.000	0.596	0.005	0.660	0.000	0.630	0.001	0.677	0.001	0.706	0.014	**0.790**	0.002
SRC	0.663	0.000	0.660	0.001	0.689	0.000	0.692	0.002	0.735	0.001	0.802	0.013	**0.875**	0.002

Table S5: Full regression results in mean squared error (MSE). Similar to the coefficient of determination R^2, the MSE suggests that Attention FP is the best model and that logP and QED are much easier to predict than docking scores.

	Ridge		Lasso		XGBoost		GP (exact)		GP (sparse)		MPNN		Attentive FP	
Target	mean	std	mean	std	mean	std	mean	std	mean	std	mean	std	mean	std
logP	0.751	0.001	0.750	0.000	0.554	0.000	0.612	0.007	0.593	0.015	0.098	0.014	**0.001**	0.000
QED	0.016	0.000	0.017	0.000	0.011	0.000	0.012	0.000	0.014	0.002	0.003	0.000	**0.001**	0.000
ADAM17	0.427	0.000	0.434	0.009	0.359	0.000	0.383	0.003	0.334	0.001	0.267	0.033	**0.188**	0.006
ESR1	0.438	0.000	0.457	0.014	0.379	0.000	0.414	0.002	0.364	0.001	0.342	0.014	**0.237**	0.002
ESR2	0.451	0.001	0.455	0.006	0.391	0.000	0.435	0.002	0.383	0.000	0.385	0.001	**0.290**	0.007
F10	0.408	0.000	0.412	0.003	0.372	0.000	0.370	0.001	0.323	0.001	0.304	0.033	**0.170**	0.001
F2	0.339	0.000	0.348	0.009	0.322	0.000	0.304	0.002	0.264	0.000	0.208	0.005	**0.124**	0.001
HSD11B1	0.675	0.000	0.676	0.001	0.497	0.000	0.537	0.004	0.456	0.002	0.446	0.027	**0.242**	0.007
IGF1R	0.376	0.000	0.384	0.009	0.331	0.000	0.347	0.002	0.301	0.001	0.284	0.033	**0.181**	0.002
JAK2	0.410	0.000	0.411	0.000	0.336	0.000	0.357	0.001	0.309	0.002	0.258	0.007	**0.158**	0.003
KIT	0.466	0.000	0.479	0.013	0.384	0.000	0.428	0.002	0.373	0.001	0.289	0.006	**0.229**	0.009
LCK	0.327	0.000	0.330	0.002	0.291	0.000	0.289	0.001	0.252	0.000	0.209	0.026	**0.109**	0.000
MAPK14	0.333	0.000	0.337	0.005	0.282	0.000	0.294	0.001	0.254	0.001	0.219	0.008	**0.152**	0.003
MAPKAPK2	0.323	0.000	0.325	0.001	0.283	0.000	0.288	0.002	0.252	0.001	0.203	0.030	**0.139**	0.010
MET	0.516	0.000	0.520	0.005	0.421	0.000	0.459	0.005	0.397	0.002	0.292	0.014	**0.245**	0.011
NR3C1	0.992	0.003	0.993	0.001	0.675	0.000	0.751	0.004	0.642	0.003	0.769	0.040	**0.331**	0.007
PARP1	0.463	0.000	0.472	0.007	0.436	0.000	0.405	0.003	0.359	0.003	0.291	0.016	**0.142**	0.003
PDE5A	0.631	0.000	0.631	0.001	0.536	0.000	0.533	0.003	0.470	0.006	0.365	0.021	**0.236**	0.005
PGR	0.707	0.002	0.704	0.001	0.611	0.000	0.661	0.007	0.571	0.000	0.630	0.090	**0.300**	0.007
PPARA	0.538	0.000	0.541	0.002	0.451	0.000	0.476	0.003	0.413	0.001	0.335	0.022	**0.226**	0.011
PPARD	0.518	0.000	0.521	0.001	0.439	0.000	0.465	0.005	0.400	0.001	0.304	0.014	**0.209**	0.004
PPARG	0.403	0.000	0.411	0.008	0.363	0.000	0.380	0.002	0.329	0.001	0.258	0.011	**0.205**	0.003
PTGS2	0.693	0.001	0.706	0.018	0.616	0.000	0.666	0.004	0.595	0.003	0.586	0.004	**0.421**	0.020
PTK2	0.385	0.000	0.393	0.003	0.322	0.000	0.339	0.003	0.297	0.002	0.246	0.013	**0.159**	0.002
PTPN1	0.383	0.000	0.387	0.004	0.326	0.000	0.354	0.001	0.310	0.001	0.282	0.013	**0.201**	0.002
SRC	0.341	0.000	0.344	0.001	0.315	0.000	0.311	0.002	0.268	0.001	0.200	0.013	**0.126**	0.002

Table S6: Full regression results in mean absolute error (MAE). Similar to the coefficient of determination R^2 and the mean squared error MSE, the MAE suggests that Attention FP is the best model and that logP and QED are much easier to predict than docking scores.

	Ridge		Lasso		XGBoost		GP (exact)		GP (sparse)		MPNN		Attentive FP	
Target	mean	std	mean	std	mean	std	mean	std	mean	std	mean	std	mean	std
logP	0.643	0.000	0.642	0.000	0.541	0.000	0.570	0.006	0.565	0.013	0.178	0.012	**0.008**	0.003
QED	0.099	0.000	0.104	0.001	0.082	0.000	0.085	0.000	0.089	0.006	0.038	0.001	**0.015**	0.001
ADAM17	0.509	0.000	0.514	0.006	0.462	0.000	0.478	0.002	0.442	0.001	0.395	0.027	**0.327**	0.005
ESR1	0.522	0.000	0.534	0.009	0.478	0.000	0.503	0.002	0.468	0.001	0.455	0.012	**0.364**	0.001
ESR2	0.524	0.001	0.527	0.004	0.480	0.000	0.511	0.001	0.475	0.000	0.479	0.001	**0.400**	0.005
F10	0.505	0.000	0.508	0.002	0.476	0.000	0.476	0.001	0.440	0.000	0.427	0.026	**0.317**	0.000
F2	0.455	0.000	0.462	0.007	0.441	0.000	0.427	0.002	0.395	0.001	0.341	0.004	**0.269**	0.002
HSD11B1	0.641	0.000	0.642	0.001	0.532	0.000	0.562	0.002	0.508	0.002	0.504	0.020	**0.340**	0.008
IGF1R	0.481	0.000	0.487	0.007	0.450	0.000	0.460	0.002	0.425	0.001	0.414	0.030	**0.323**	0.002
JAK2	0.493	0.000	0.493	0.000	0.444	0.000	0.457	0.001	0.420	0.002	0.383	0.008	**0.288**	0.003
KIT	0.534	0.000	0.542	0.008	0.480	0.000	0.509	0.001	0.472	0.001	0.413	0.005	**0.365**	0.008
LCK	0.445	0.000	0.447	0.001	0.418	0.000	0.417	0.001	0.386	0.001	0.345	0.023	**0.254**	0.001
MAPK14	0.452	0.000	0.455	0.003	0.415	0.000	0.423	0.001	0.391	0.001	0.361	0.007	**0.299**	0.004
MAPKAPK2	0.440	0.000	0.442	0.001	0.410	0.000	0.410	0.001	0.379	0.001	0.339	0.030	**0.278**	0.011
MET	0.569	0.000	0.572	0.003	0.510	0.000	0.531	0.003	0.490	0.001	0.415	0.010	**0.377**	0.009
NR3C1	0.773	0.001	0.773	0.000	0.603	0.000	0.655	0.002	0.592	0.002	0.657	0.014	**0.390**	0.008
PARP1	0.533	0.000	0.539	0.004	0.513	0.000	0.491	0.001	0.457	0.003	0.399	0.012	**0.282**	0.003
PDE5A	0.611	0.000	0.612	0.000	0.558	0.000	0.554	0.002	0.514	0.004	0.445	0.015	**0.348**	0.005
PGR	0.666	0.001	0.666	0.001	0.612	0.000	0.643	0.004	0.596	0.000	0.621	0.051	**0.394**	0.008
PPARA	0.574	0.000	0.577	0.002	0.520	0.000	0.532	0.001	0.490	0.001	0.436	0.016	**0.343**	0.013
PPARD	0.566	0.000	0.568	0.001	0.519	0.000	0.532	0.003	0.488	0.001	0.420	0.012	**0.344**	0.003
PPARG	0.501	0.000	0.507	0.006	0.472	0.000	0.483	0.002	0.446	0.001	0.389	0.010	**0.346**	0.003
PTGS2	0.656	0.000	0.663	0.009	0.610	0.000	0.640	0.002	0.602	0.002	0.596	0.004	**0.479**	0.011
PTK2	0.484	0.000	0.490	0.002	0.438	0.000	0.448	0.002	0.414	0.002	0.374	0.009	**0.293**	0.001
PTPN1	0.485	0.000	0.488	0.003	0.443	0.000	0.465	0.001	0.432	0.001	0.411	0.011	**0.346**	0.003
SRC	0.457	0.000	0.460	0.001	0.436	0.000	0.435	0.001	0.400	0.001	0.337	0.012	**0.270**	0.002

C.2 Virtual Screening

C.2.1 Additional task details.

Training. All training details are identical to those presented in Section C.1.

ZINC dataset. Because the ZINC dataset grows over time, a copy of the dataset was downloaded from `https://zinc20.docking.org/` in July 2021 to be used as the standard dataset for this task. It contained 997597004 SMILES strings. There were 56606 items in common between ZINC and the training set, representing 22% of the training set.

Enrichment factor calculation. Because the true docking scores of all compounds in the ZINC dataset are too expensive to calculate, the exact cutoff for the top 0.1% of the dataset is unknown. To estimate it, we selected a random subset of ZINC20 of size 100k for each target and calculated the scores with DOCKSTRING. The top 0.1% of this subset was used as an estimate of the true 0.1% cutoff.

Threshold for enrichment factor. The threshold of 0.1% was chosen for two reasons. First, it has been given as the approximate hit rate of high-throughput screening.[4] Second, if the threshold were higher (say top 1%), the task would not be as challenging, and the differences in methods might not be as apparent. Third, the 0.1% threshold is estimated from a sample size of 100,000, making it the docking score of the 100th best molecule in the sample. If the percentile were much lower, the estimate of the cutoff value might be unreliable.

C.2.2 Results.

How good are the docking scores? For KIT, ridge finds one, and Attentive FP finds 35 molecules with docking scores lower than the lowest in the training set. For PARP1, ridge finds one, and Attentive FP finds ten molecules with docking scores lower than the lowest in

the training set. For PGR, ridge and Attentive FP find zero molecules with docking scores lower than the lowest in the training set.

How do the results depend on the threshold for active molecules? If the threshold is increased (e.g., top 1%), the results are qualitatively the same, although the quantitative differences are less pronounced. Lowering the threshold has the opposite effect.

C.3 *De Novo* Molecular Design

C.3.1 Baseline methods.

Rationale for choice of baselines. We selected the methods to represent two broad classes of algorithms used in previous work. Genetic algorithms are commonly used for molecular design: the graph genetic algorithm[5] was chosen due to its strong performance in the GuacaMol baselines,[6] while the SELFIES genetic algorithm was chosen due to its simplicity. We believe these algorithms are representative of a broader class of model-free, exploratory algorithms. Bayesian optimization with a Gaussian process (GP) was chosen because Bayesian optimization is widely regarded as a high-performing optimization technique when the number of function evaluations is limited.[7] GPs are the most common model used in Bayesian optimization. The acquisition functions chosen are the most commonly employed acquisition functions as far as we are aware. We believe that GP-BO represents a broader class of model-based algorithms that can be used for both exploration and exploitation. Finally, random ZINC is an important trivial baseline which acts as a lower bound for acceptable performance for an algorithm.

Maximization or minimization? Although most of the objectives in Section 3.3.3 are minimization objectives, our code was designed for maximization. Therefore all minimization objectives are multiplied by -1 and maximized. Technical descriptions in the remainder of this section therefore correspond to maximization.

Reinforcement learning. We omitted any baseline methods based on reinforcement learning. This is because, to our knowledge, previously reported policy reinforcement learning methods required many more than 5000 objective function evaluations to achieve reasonable performance (e.g., Ref. 8). Reinforcement learning will be explored in future version of this manuscript.

C.3.2 Results

Rank	Objective	logP	Molecular Weight	HBA	HBD	QED
1	43.268	43.268	3069.380	36	0	0.014
2	42.629	42.629	3190.526	33	0	0.014
3	42.486	42.486	3157.732	33	0	0.014
4	42.439	42.439	3068.325	36	0	0.014
5	41.997	41.997	3188.366	37	0	0.014
6	41.960	41.960	3193.075	33	0	0.014

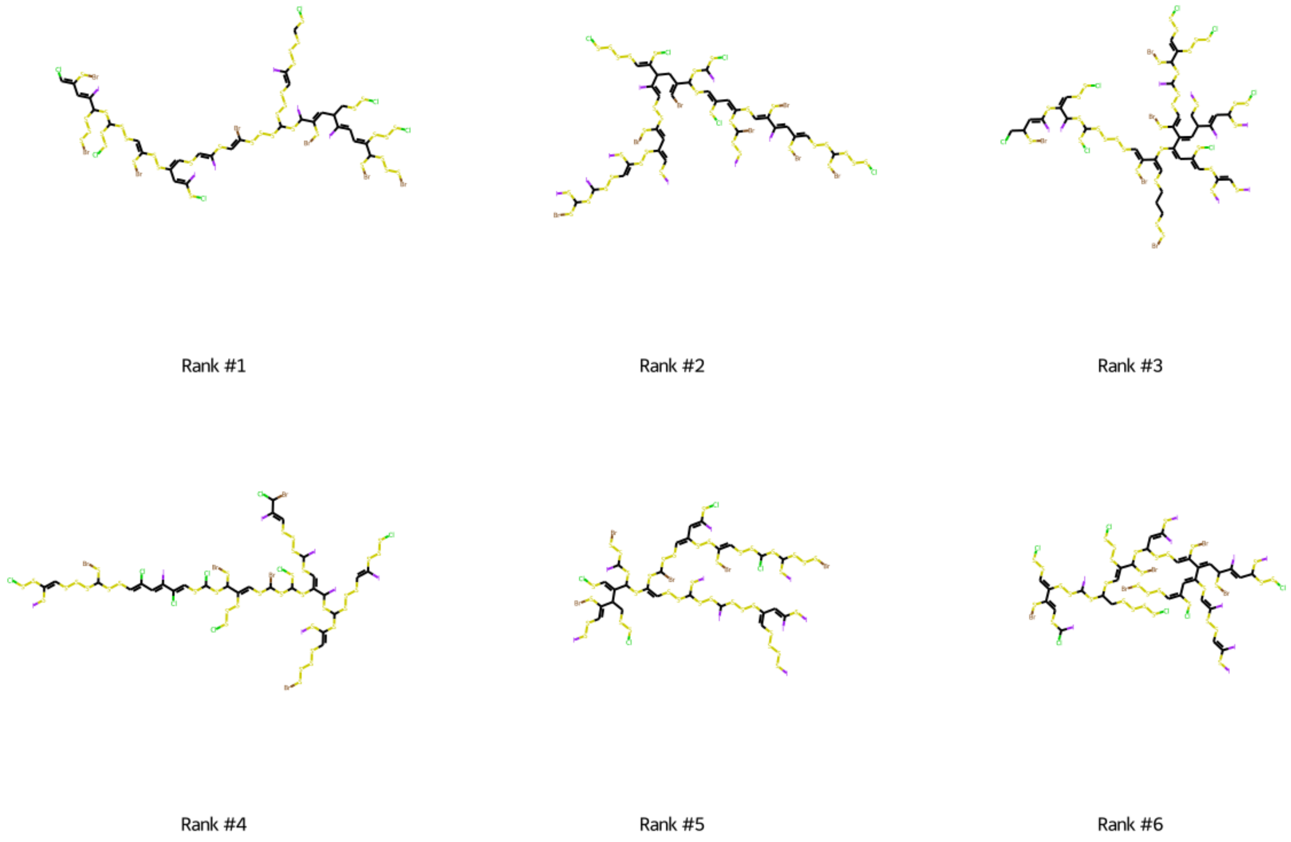

Figure S1: Best molecules for logP optimization.

C.4 Analysis of *de novo* Kinase Inhibitors

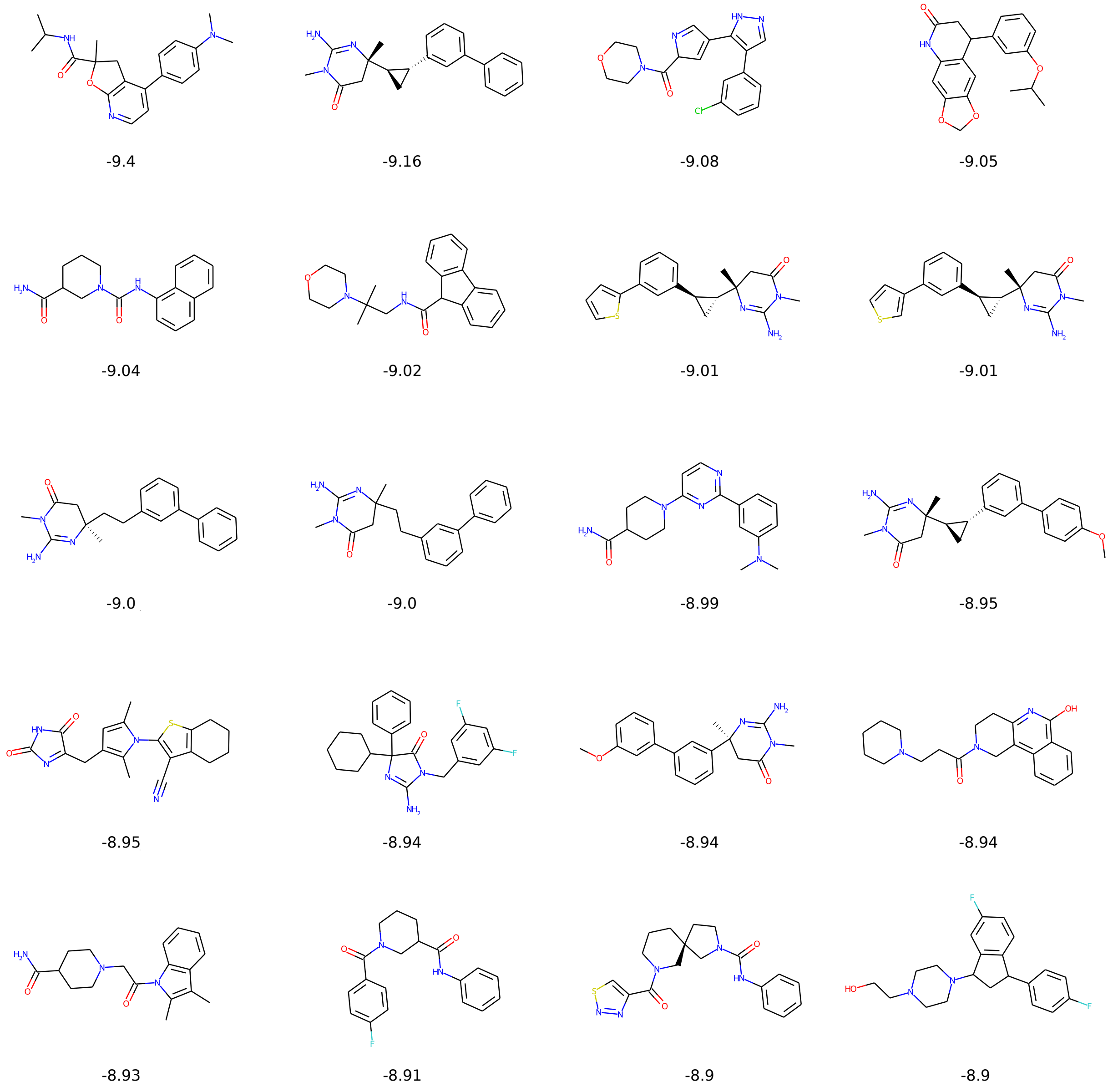

Figure S2: Training molecules with the highest scores in the Selective JAK2 task.

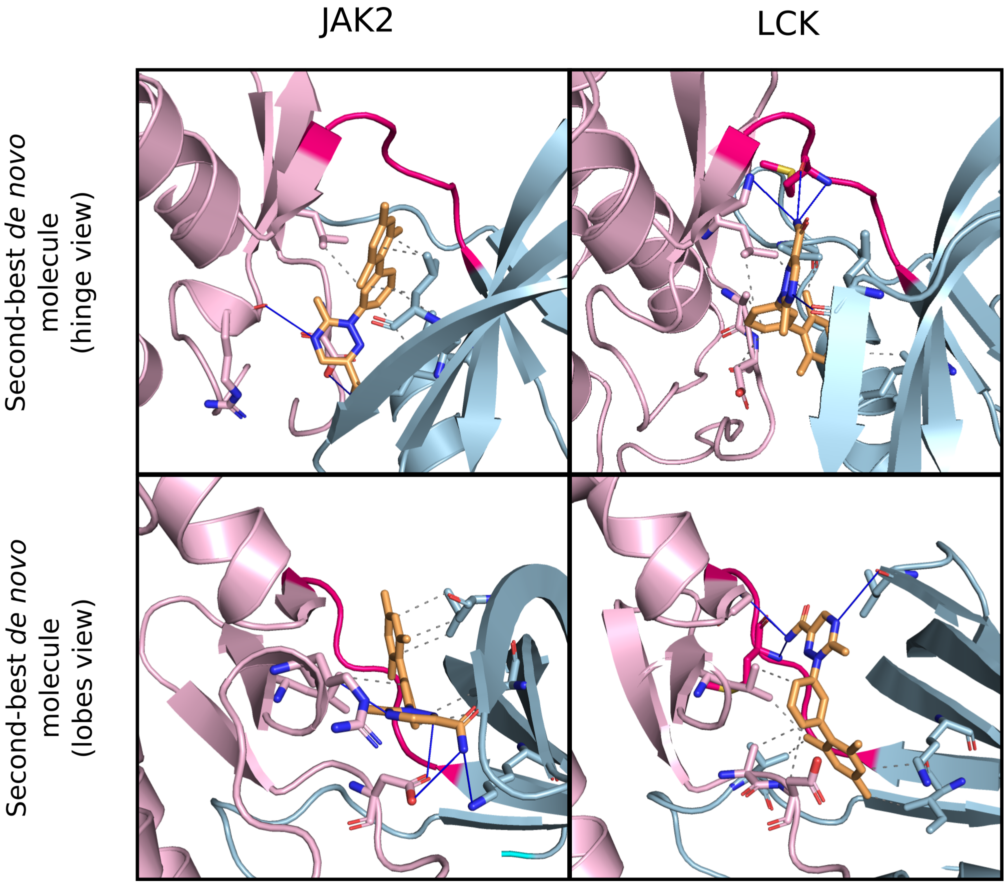

Figure S3: Pose analysis of the second-best *de novo* molecule in the Selective JAK2 task. Both poses in JAK2 and LCK were almost identical to the poses of the best *de novo* molecule (Figure 13).

D Computational Details

D.1 Docking Run Time

Average DOCKSTRING docking run times are shown in Table S7.

D.2 Dataset

Docking scores were computed in a cluster environment using the resources of the Cambridge Service for Data Driven Discovery (CSD3). Each score was calculated using a single core of a Intel Xeon Skylake, 2.6GHz 16-core, on a node with 3.42MB of RAM. In addition to the more than 15 million docking scores in the DOCKSTRING dataset, we also computed scores for assessing the quality of each target, and for determining the optimal search box sizes. In total, the preparation and computation of the dataset required more than 500k CPU hours.

D.3 Baselines

The regression baselines were relatively inexpensive. Each run of lasso, ridge regression, XGBoost, and GPs took under 1h on a single machine with 6 CPUs. MPNN and Attentive FP methods each took around 2h on a single machine with a NVIDIA 2080 Ti GPU. Training of the virtual screening models was identical to the regression. Prediction on ZINC took around 10 CPU hours for ridge regression and 1500 CPU hours for Attentive FP. The molecular optimization tasks took between 24-72 hours to run for all methods (on a machine with 8 CPUs) depending on the optimization trajectory and the number of calls to DOCKSTRING required to evaluate each objective. In total, we estimate that all benchmark tasks collectively required about 20k CPU hours.

Table S7: Mean (and standard deviation) of docking times for all targets with DOCKSTRING, averaged over 50 ligands from the DOCKSTRING dataset. Results are shown for different numbers of CPUs. The same set of 50 ligands was used for every target. Continued in Table S8.

Target	1 CPU	2 CPUs	4 CPUs	8 CPUs	16 CPUs	32 CPUs
ABL1	123.5 (75.9)	59.2 (33.9)	30.7 (17.2)	21.2 (11.5)	15.6 (8.5)	14.3 (7.6)
ACHE	93.6 (58.4)	53.7 (30.9)	28.3 (14.5)	17.4 (7.6)	14.1 (6.4)	14.2 (6.5)
ADAM17	101.9 (61.0)	43.8 (25.6)	26.3 (17.0)	14.8 (6.9)	11.8 (5.5)	11.7 (5.4)
ADORA2A	92.3 (58.2)	48.7 (31.3)	26.1 (15.1)	15.5 (8.1)	12.4 (6.7)	12.3 (6.6)
ADRB1	97.2 (62.2)	50.0 (31.8)	30.6 (19.7)	18.7 (10.3)	14.7 (8.8)	14.6 (8.7)
ADRB2	104.1 (65.9)	53.7 (33.0)	30.4 (18.3)	19.3 (10.6)	15.7 (9.2)	15.7 (9.0)
AKT1	74.8 (51.3)	57.1 (37.1)	29.1 (18.4)	18.1 (10.0)	15.4 (8.9)	15.1 (8.5)
AKT2	95.9 (60.9)	48.5 (29.5)	23.0 (12.4)	16.7 (7.9)	13.0 (6.2)	13.0 (6.2)
AR	86.6 (55.8)	57.7 (36.4)	31.1 (17.9)	18.9 (9.8)	16.2 (9.0)	15.7 (8.6)
BACE1	88.4 (52.8)	48.5 (32.4)	25.0 (15.2)	13.7 (6.3)	11.7 (5.2)	11.5 (5.1)
CA2	100.2 (64.2)	56.7 (38.6)	28.6 (16.9)	17.0 (9.2)	14.5 (8.2)	16.4 (9.2)
CASP3	88.0 (54.6)	45.9 (28.4)	27.5 (16.1)	14.6 (7.4)	11.6 (5.8)	11.7 (5.9)
CDK2	68.9 (41.9)	44.6 (26.7)	24.4 (13.7)	14.2 (7.0)	11.6 (5.7)	11.5 (5.6)
CSF1R	91.2 (55.6)	44.2 (26.4)	24.3 (13.5)	14.8 (7.5)	11.7 (5.9)	11.8 (5.8)
CYP2C9	95.2 (59.4)	53.7 (31.6)	29.7 (17.5)	16.6 (8.2)	14.5 (7.6)	15.7 (8.0)
CYP3A4	85.9 (51.4)	48.6 (32.5)	25.6 (15.6)	15.3 (8.0)	12.8 (6.9)	12.6 (6.9)
DHFR	83.6 (50.5)	44.4 (26.8)	23.6 (13.2)	14.9 (7.7)	12.0 (6.2)	12.0 (6.1)
DPP4	103.3 (61.4)	60.0 (37.7)	28.0 (14.6)	16.6 (7.4)	13.6 (6.3)	14.0 (6.4)
DRD2	102.7 (68.4)	56.4 (39.2)	29.8 (18.2)	18.3 (10.1)	16.1 (9.4)	16.1 (9.2)
DRD3	88.6 (59.8)	50.5 (36.3)	26.4 (16.2)	16.5 (8.8)	13.4 (7.2)	13.5 (7.2)
EGFR	83.8 (52.3)	50.0 (30.6)	26.7 (14.8)	16.6 (8.0)	13.4 (6.6)	13.5 (6.6)
ESR1	95.8 (58.0)	46.4 (27.4)	25.6 (14.4)	15.5 (7.7)	12.3 (6.3)	12.3 (6.1)
ESR2	89.7 (54.0)	47.1 (28.1)	25.2 (13.4)	15.3 (7.6)	12.2 (6.0)	12.2 (6.0)
F10	93.2 (56.8)	50.7 (37.8)	25.2 (14.3)	15.5 (7.8)	12.3 (6.0)	12.2 (6.1)
F2	89.5 (53.6)	46.4 (29.8)	23.6 (13.3)	13.2 (6.2)	11.3 (5.4)	11.4 (5.3)
FGFR1	285.8 (198.4)	50.6 (33.8)	27.7 (17.4)	16.5 (9.1)	13.4 (7.7)	13.4 (7.6)
GBA	102.5 (65.9)	60.5 (43.4)	30.7 (16.8)	18.6 (9.1)	15.4 (8.1)	15.4 (7.9)
HMGCR	266.0 (165.0)	47.8 (28.4)	27.2 (14.7)	16.0 (7.5)	12.7 (6.1)	12.8 (6.1)
HSD11B1	101.4 (64.8)	51.7 (37.0)	27.7 (16.4)	16.4 (8.6)	14.0 (7.4)	13.9 (7.3)
HSP90AA1	258.2 (159.9)	47.0 (28.1)	23.2 (13.2)	16.4 (8.1)	13.1 (6.4)	13.1 (6.4)
IGF1R	91.5 (55.1)	45.8 (26.7)	25.0 (13.6)	15.7 (9.2)	12.2 (6.1)	12.0 (6.0)

Table S8: Table S7 continued.

Target	1 CPU	2 CPUs	4 CPUs	8 CPUs	16 CPUs	32 CPUs
JAK2	91.4 (56.7)	45.5 (27.5)	24.9 (13.6)	15.1 (7.4)	11.8 (5.9)	11.8 (5.7)
KDR	104.1 (64.5)	55.9 (37.3)	26.9 (16.0)	16.2 (8.4)	13.9 (7.3)	14.0 (7.3)
KIT	254.2 (157.7)	46.0 (27.7)	25.2 (14.2)	15.4 (7.4)	12.4 (6.0)	12.4 (5.9)
LCK	85.2 (51.5)	42.4 (24.9)	22.8 (12.4)	14.0 (6.7)	11.1 (5.2)	11.1 (5.1)
MAOB	97.6 (59.1)	52.5 (30.8)	36.1 (20.5)	20.8 (10.5)	17.2 (8.6)	17.2 (8.5)
MAP2K1	91.4 (58.8)	52.0 (31.8)	28.3 (16.7)	17.0 (8.8)	13.7 (7.2)	13.5 (7.0)
MAPK1	93.5 (58.3)	53.6 (38.3)	25.2 (14.6)	15.4 (8.1)	12.3 (6.2)	12.5 (6.3)
MAPK14	92.2 (58.8)	49.8 (39.4)	25.7 (15.5)	15.7 (8.7)	12.6 (7.1)	12.6 (7.0)
MAPKAPK2	88.3 (52.7)	53.7 (32.8)	25.0 (14.0)	15.2 (7.5)	11.9 (6.0)	11.9 (5.8)
MET	100.5 (62.3)	50.5 (30.0)	27.5 (15.0)	16.4 (7.7)	13.5 (6.5)	13.3 (6.5)
MMP13	86.2 (54.6)	49.2 (30.5)	23.9 (13.8)	14.2 (7.1)	11.3 (5.8)	11.5 (5.8)
NOS1	94.1 (62.0)	60.1 (39.7)	28.9 (17.1)	17.3 (8.5)	14.2 (7.1)	14.4 (7.2)
NR3C1	105.4 (65.2)	52.6 (32.5)	30.5 (17.6)	18.7 (9.5)	15.9 (8.8)	15.7 (8.5)
PARP1	82.3 (48.0)	48.4 (31.1)	24.7 (13.3)	14.7 (6.9)	11.7 (5.6)	11.8 (5.5)
PDE5A	85.2 (48.9)	51.0 (29.2)	25.5 (13.6)	15.2 (7.3)	12.1 (5.8)	11.9 (5.7)
PGR	89.0 (60.4)	61.7 (38.3)	31.9 (19.5)	19.1 (10.3)	16.7 (9.5)	16.1 (9.1)
PLK1	93.7 (58.8)	49.7 (38.1)	25.8 (14.7)	15.5 (8.1)	12.4 (6.6)	12.4 (6.4)
PPARA	99.9 (63.1)	56.1 (34.7)	27.4 (15.8)	16.7 (8.6)	13.6 (7.4)	13.7 (7.5)
PPARD	89.0 (55.4)	52.9 (32.1)	25.9 (15.1)	16.1 (8.3)	13.7 (7.1)	13.2 (6.8)
PPARG	87.5 (54.1)	49.0 (29.8)	23.8 (13.4)	14.7 (7.0)	11.6 (5.6)	11.6 (5.5)
PTGS2	99.2 (61.1)	58.2 (35.5)	29.7 (16.3)	17.8 (8.4)	14.8 (7.4)	14.6 (7.3)
PTK2	256.9 (163.8)	46.3 (29.0)	25.5 (15.9)	15.4 (8.3)	12.2 (6.6)	12.3 (6.6)
PTPN1	76.9 (46.9)	55.6 (36.1)	25.6 (15.0)	15.0 (7.0)	12.5 (6.1)	12.1 (5.7)
REN	85.9 (52.1)	43.8 (26.9)	24.1 (12.8)	14.6 (6.8)	11.7 (5.3)	11.5 (5.3)
ROCK1	69.3 (42.9)	52.8 (37.8)	24.9 (14.2)	14.6 (6.7)	16.3 (7.6)	11.7 (5.4)
SRC	90.8 (58.1)	47.3 (29.8)	25.3 (15.2)	14.5 (7.5)	12.5 (6.7)	12.5 (6.5)
THRB	101.5 (63.6)	55.2 (38.9)	27.1 (15.6)	16.0 (8.2)	14.4 (7.7)	14.3 (7.6)

E Maintenance Plan

The DOCKSTRING Python package will be hosted on GitHub and actively developed. Since it is vital to ensure that the package is compatible with our dataset (i.e. that it can be used to generate the same numbers), we will perform frequent testing to ensure that future changes do not change the numerical output of the package. In particular, we will work to ensure that the package still functions even when new versions of the major dependencies (i.e. `rdkit`, `openbabel`) are released. If breaking changes are required to implement new features, we will to split the project into a different package (e.g. DOCKSTRING2) to preserve the original version.

The DOCKSTRING dataset is fixed and will continue to be hosted on `https://dockstring.github.io` so that its standardized form can be accessed by researchers. We are interested in expanding the dataset in the future to include more targets / ligands, and plan to follow the model of ZINC[9,10] by releasing updated versions of the dataset as separate re-numbered datasets (e.g. DOCKSTRING2022).

The code for DOCKSTRING benchmarks will be hosted on GitHub upon publication. We also plan to host a public leader board and list of publications that use DOCKSTRING's benchmarks. The benchmarks presented in this work are only 3 of many possible benchmarks that DOCKSTRING enables. In future work, we plan to develop and promote other benchmarks, starting with tasks for transfer learning, meta-learning and few-shot learning. These will likely be released with future publications and linked to on the DOCKSTRING website.

References

(1) Lipinski, C. A.; Lombardo, F.; Dominy, B. W.; Feeney, P. J. Experimental and Computational Approaches to Estimate Solubility and Permeability in Drug Discovery and Development. *Adv. Drug Delivery Rev.* **2001**, *46*, 3–26.

(2) Yusof, I.; Segall, M. D. Considering the impact drug-like properties have on the chance of success. *Drug Discovery Today* **2013**, *18*, 659–666.

(3) Wishart, D. S.; Knox, C.; Guo, A. C.; Shrivastava, S.; Hassanali, M.; Stothard, P.; Chang, Z.; Woolsey, J. DrugBank: a comprehensive resource for in silico drug discovery and exploration. *Nucleic Acids Res.* **2006**, *34*, D668–D672.

(4) Bender, A.; Bojanic, D.; Davies, J. W.; Crisman, T. J.; Mikhailov, D.; Scheiber, J.; Jenkins, J. L.; Deng, Z.; Hill, W. A. G.; Popov, M.; Jacoby, E.; Glick, M. Which aspects of HTS are empirically correlated with downstream success? *Curr. Opin. Drug Discovery Dev.* **2008**, *11*, 327–337.

(5) Jensen, J. H. A graph-based genetic algorithm and generative model/Monte Carlo tree search for the exploration of chemical space. *Chem. Sci.* **2019**, *10*, 3567–3572.

(6) Brown, N.; Fiscato, M.; Segler, M. H.; Vaucher, A. C. GuacaMol: benchmarking models for de novo molecular design. *J. Chem. Inf. Model.* **2019**, *59*, 1096–1108.

(7) Shahriari, B.; Swersky, K.; Wang, Z.; Adams, R. P.; De Freitas, N. Taking the human out of the loop: A review of Bayesian optimization. *Proceedings of the IEEE* **2015**, *104*, 148–175.

(8) You, J.; Liu, B.; Ying, R.; Pande, V.; Leskovec, J. Graph convolutional policy network for goal-directed molecular graph generation. Proceedings of the 32nd International Conference on Neural Information Processing Systems. 2018; pp 6412–6422.

(9) Irwin, J. J.; Sterling, T.; Mysinger, M. M.; Bolstad, E. S.; Coleman, R. G. ZINC: a free tool to discover chemistry for biology. *J. Chem. Inf. Model.* **2012**, *52*, 1757–1768.

(10) Irwin, J. J.; Tang, K. G.; Young, J.; Dandarchuluun, C.; Wong, B. R.; Khurelbaatar, M.; Moroz, Y. S.; Mayfield, J.; Sayle, R. A. ZINC20—a free ultralarge-scale chemical database for ligand discovery. *J. Chem. Inf. Model.* **2020**, *60*, 6065–6073.
